# Psychological Interventions for Young People With Psychotic Disorders: A Systematic Review

**DOI:** 10.3389/fpsyt.2022.859042

**Published:** 2022-03-24

**Authors:** Vera Gergov, Branka Milic, Henriette Löffler-Stastka, Randi Ulberg, Eleni Vousoura, Stig Poulsen

**Affiliations:** ^1^Department of Psychology and Logopedics, Faculty of Medicine, University of Helsinki, Helsinki, Finland; ^2^Department of Psychoanalysis and Psychotherapy, Medical University of Vienna, Vienna, Austria; ^3^Institute of Clinical Medicine, University of Oslo, Oslo, Norway; ^4^Department of Psychiatry, Diakonhjemmet Hospital, Oslo, Norway; ^5^Department of Psychiatry, University of Athens, Athens, Greece; ^6^Department of Psychology, University of Copenhagen, Copenhagen, Denmark

**Keywords:** psychotic disorders, psychotherapy, systematic review, adolescent, young adult

## Abstract

**Background:**

Psychotic disorders are commonly accompanied by intense psychological burden, and psychological interventions are usually needed in order to reduce the symptoms and help in maintaining or improving the level of psychological and social functioning after the onset of psychosis. The evidence-base for treating young people at risk for psychosis and adults with psychotic disorders is accumulating. Yet, pervasive systematic literature reviews that would include patients from the full age range being the most essential period for the risk of developing a psychotic disorder, a wide range of psychological interventions, and various types of clinical trials, have been lacking. The aim of this systematic review is to fill the gap by presenting the current research evidence from clinical trials on the effectiveness of psychological interventions for treating young people (12–30) with psychotic disorders.

**Methods:**

A systematic search was conducted in PubMed and PsycINFO followed by a 3-step screening process based on the PICOS strategy. Risk of bias of the included studies was assessed by the Mixed Methods Appraisal Tool (MMAT). Extracted data from the included studies is reported using a narrative synthesis.

**Results:**

Of the 1,449 publications screened, 40 from 25 studies were included in the review. Of these, 10 studies reported results from cognitive or behavioral therapy, nine from cognitive remediation therapy (CRT), and six from other types of therapies (i.e., integrative interventions combining psychoeducation and family/group interventions). All but one study found the target interventions to be effective, but the results mostly did not differ significantly from the control conditions in reducing symptoms and improving functioning, preventing relapses and hospitalization, or improving psychological or family variables. The most consistent findings were from CRT, showing more improvement in cognitive functioning compared to control conditions while not being superior in reducing symptom severity. Integrative interventions might be effective in treating young people suffering from psychotic disorders.

**Conclusion:**

There is some evidence that psychological interventions are effective for young people with psychotic disorders. However, with regard to symptom severity, psychotherapy does not outperform control conditions, and the results do not strongly favor any specific type of treatment.

**Systematic Review Registration:**

[https://www.crd.york.ac.uk/prospero/display_record.php?ID=CRD42020166756], identifier [CRD42020166756].

## Introduction

Psychiatric disorders constitute a global major health problem, due to their high prevalence, the related functional and symptomatic deficits, and the direct and indirect effects on patients’ socio-economic circumstances and social environments (1). Disorders of the psychotic spectrum are defined by typical and profound “distortions of thinking and perception and affects that are inappropriate or blunted” (2). The DSM-5 characterizes psychotic disorders by deviations in the following aspects: negative symptoms (e.g., social anhedonia, reduced emotional expression, impaired functioning), disorganized thinking, hallucinations, delusions, and/or abnormal motor behavior (3). The estimated annual prevalence of psychotic disorders is 2.6% (4). The prevalence rises from childhood to adolescence, the highest risk age being from 15 to 30 (5). Especially in the early stages of psychosis, the differentiation between non-specific symptoms that are typical for adolescents in several mental disorders, and the prodromal symptoms indicating psychosis, is challenging as they include the same symptoms (e.g., reduced ability to concentrate, decrease in motivation, sleep disturbance, depression, anxiety, cognitive and social impairment, decreased tolerance for stress) (6). The diagnosis can often be confirmed only after a longer time of follow-up. In general, less than 24% of adolescents considered to be at high risk for psychosis eventually develop psychosis (7).

Due to its multimodal consequences, psychosis is commonly accompanied by intense psychological strain. As the individual risk for developing psychosis has multifactorial explanations, the specification of an individualized treatment strategy should also take multiple factors into account. Until recently, it has not been possible to define a coherent list of clinical, psychological, and social factors determining the individual likelihood to benefit from treatment, as the evidence on variables predicting treatment outcome is scarce and studies show contradictory results. The most frequently reported predictors of poor treatment outcomes are premorbid difficulties, symptom severity (especially of negative symptoms) at baseline, and duration of untreated psychosis (DUP) (8, 9), suggesting that early intervention is an important clinical goal. Early and more assertive interventions in non-responders can probably improve the prognosis of psychosis (10).

Generally, a combination of pharmacological treatment and psychological interventions is the first-line recommendation for treating psychosis (11, 12). The combination has been found to be the most effective in treating symptoms, improving functional outcomes, increasing recovery rates, and reducing hospital admission rates. At the same time, there is an ongoing debate in the field about the appropriateness and efficacy of different treatment options for schizophrenia (13). As pharmacological therapies have been criticized for burdensome side-effects, high non-response, and non-compliance rates (13, 14), it is useful to consider and improve the effectiveness of psychological interventions on the specific outcomes related to psychotic disorders. Psychological interventions aim to minimize the harm caused by the mental disorder and advance age-appropriate psychological development and promote social competence. Importantly, psychological interventions may also improve the patient’s and relatives’ commitment to overall treatment. As mentioned, early referral to specialist mental health services is critical, so that appropriate interventions can be provided to improve outcomes and long-term outlook. The younger the patient is, and the more severe the symptoms are, the more important it is to collaborate not just with the individual, but also with the family and other network members (15). Providing specialized early intervention to treat recent−onset psychosis is likely to have benefits: more people continue with their treatment, and the number of people who recover increases in comparison to treatment as usual (TAU) (16). Psychoeducation is also suggested to be an important part of the treatment of psychotic disorders (17). According to several national guidelines and best practice recommendations, psychoeducational single or multiple family groups are the gold standard in treatment (18).

While the effects of specific forms of psychotherapy (e.g., psychodynamic, cognitive and behavioral, humanistic, and systemic therapies) are known in general (19), and the evidence-base for treating young people at risk for psychosis (6) and adults with psychotic disorders (11, 16, 20) are accumulating, systematic reviews on the psychotherapy outcome, especially in young patients with psychotic disorders, are still very few. In the recent meta-analysis by Datta et al. (21), cognitive remediation therapy (CRT), psychoeducation, family therapy, and group psychotherapy were found to be useful for adolescents with psychotic disorders. However, the review included only randomized controlled trials (RCTs) for patients with psychotic disorders, aged 13–17 years, resulting in only seven included studies with a variety of psychological interventions. Most results suggested little or no effect of the target interventions compared to control treatments, leaving the evidence on the effectiveness of psychological interventions for this specific population to be limited.

In order to have a clearer view on which specific ingredients in treatment are more likely to provide help for the patients at an early stage of psychotic disorders, the knowledge on psychological intervention studies with psychotic young patients has to be reviewed more broadly. This means that a review could preferably also include non-randomized clinical studies or studies with a wider age range.

### Aims of the Study

The aim of this systematic review is to present the research evidence from clinical trials on the effectiveness of psychological interventions for treating young people with psychotic disorders. The review focuses mainly on the clinically relevant outcomes, such as symptom reduction or remission, hospitalization, and improvements in occupational, social, and cognitive functioning, and reporting the between-group effects.

## Methods

### Search Strategy

The study was conducted in the European Network on Individualized Psychotherapy Treatment of Young People with Mental Disorders (TREATme; CA 16102) funded by the European Cooperation in Science and Technology (COST), through Horizon 2020. It is a part of a larger ongoing study aiming to carry out a number of systematic literature reviews on psychotherapeutic interventions among young people with mental disorders. The overall protocol for conducted systematic literature reviews is registered in PROSPERO (CRD42020166756) and described in Vousoura et al. (22).

In this study, a systematic literature search following the Preferred Reporting Items for Systematic Reviews and Meta-Analyses (PRISMA) guidelines (23), was conducted on the PubMed and PsycINFO databases with no publication year limitation, and a final update on 22 April 2021. The search aimed to identify studies assessing the effectiveness of psychological interventions for adolescents and young adults aged between 12 and 30 diagnosed with psychotic disorders. The search strings were formed following the PICOS (population, intervention, comparison, outcome, and study design) strategy (24) by combining search terms for (i) psychotic disorders, (ii) psychological interventions, and (iii) study type described in Table 1. The controlled descriptors (i.e., PubMed MeSH terms, PsycINFO thesaurus) and their synonyms (keywords) were verified in each database, and search terms were combined with the Boolean operator “and” and “or.” To identify relevant studies for the specific age group targeted, the age filters for “adolescents” and “young adults” were added. The filter for study type, including “clinical study” OR “comparative study” in PubMed and “clinical case study” OR “clinical trial” OR “empirical study” OR “treatment outcome” in PsycINFO was added to identify all types of clinical trials. The final search string was formed by one researcher (VG) in collaboration with information specialists (for detailed database search strings, see Supplementary Files 1A,B).

**TABLE 1:** The PICOS strategy used to form the search strings for the systematic database searches.

P - Population	Adolescents (13–18 years) and young adults (18–29 years) with psychotic disorders.
	**Keywords:**
	Schizophrenia Spectrum and Other Psychotic Disorders; Psychotic disorder; Psychosis; Psychoses; Schizophrenia; Schizoaffective; Schizophreniform; Reactive psychosis; Reactive psychoses
	**Filters:**
	adolescent OR young adult
I - Intervention	Psychological interventions defined as well-known psychotherapy approaches and other psychosocial interventions previously shown promising evidence on treating psychosis. At least one treatment condition involved in the study.
	**Keywords:**
	Psychotherapy; Psychotherapeutic treatment; Psychotherapeutic intervention; Psychological therapy; Psychological treatment; Psychological intervention; Psychosocial therapy; Psychosocial treatment; Psychosocial intervention; Supportive therapy Supportive treatment; Counseling; Counseling; Motivational interviewing; Psychoeducation; Psychoeducational; Cognitive therapy; Cognitive analytic therapy; Behavioral therapy; Behavioral therapy; CBT; Psychoanalysis; Psychodynamic therapy; Psychoanalytic therapy; Dynamic therapy; Transference focused (therapy); Mentalization based (therapy); Metacognitive therapy; Interpersonal therapy; Interpersonal and social rhythm therapy; Schema therapy; Schema-focused therapy; Acceptance and Commitment Therapy; Acceptance based (therapy); Problem solving therapy; Problem solving treatment; Insight oriented therapy; Rational emotive; Solution focused therapy; Family therapy; Family systems therapy; Parenting intervention; Parent management training; Group therapy; Mind-Body Therapy; Art Therapy; Dance Therapy; Music Therapy; Play Therapy; Expressive therapy; Cognitive remediation; Cognitive training; Behavioral activation; Behavioral activation; Behavior activation; Behavioral weight control; Behavioral weight control; Applied behavior analysis; Applied behavior analysis; Attention bias modification; Exposure and response prevention; Exposure therapy; Systematic Desensitization; Eye movement desensitization reprocessing; EMDR; Psychology biofeedback; Hypnosis; Mindfulness; Relaxation
C - Comparison	No intervention or usual care is required as a comparative treatment.
O - Outcome	Quantitative studies including pre- and post-treatment measurement points published in peer-review journals. Outcome should be clinically relevant and directed to the target diagnosis.
S – Study design	Clinical outcome trials such as RCTs, controlled trials, empirical trials, naturalistic setting and case studies are included.
	**Filters:**
	Clinical Trial OR Comparative study

### Eligibility Criteria and Study Selection

The inclusion criteria based on the PICOS strategy were that the study had to be (i) a clinical outcome study (ii) with at least one treatment condition involved (i.e., a psychological intervention of any length or orientation), (iii) for adolescents or young adults aged 12–30 years, (iv) with psychotic disorder, (v) as determined by DSM-, ICD-, or other diagnostic criteria or high level of symptoms on at least one relevant self-report measure [e.g. Brief Psychiatric Rating Scale (BPRS) (25), The Positive and Negative Syndrome Scale (PANNS) (26)]. In addition, the study had to be published in a peer-reviewed journal.

**FIGURE 1:**
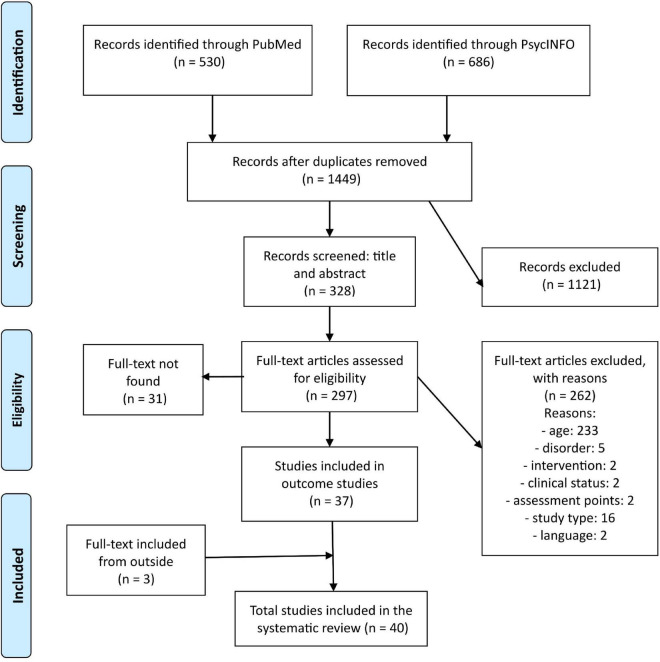
PRISMA flow diagram detailing the number of studies retained for analysis according to screening steps.

A PRISMA flow diagram detailing the number of studies retained for analysis according to screening steps is presented in Figure 1. The systematic search was conducted in PubMed and PsycINFO by one researcher (VG) and replicated independently by two researchers (EV, SP) in order to cross-check the results. The results were combined, and duplicates were removed. Next, three independent researchers (VG, HL-S, BM) started a three-step screening process. In the first stage, all titles were screened against the previously described inclusion criteria to verify whether the study was a psychological intervention program for patients with psychosis. At the second stage, the abstracts were screened against the previously described inclusion criteria and in addition, it was assessed (i) if the participants were in the age range of 12 to 30 years (ii) if it was an outcome study, (iii) published in peer-review journals, and that (iv) the full-text was available in English. In the case that a decision of whether the article should be included could not be reached based solely on the title and abstract, the study was included for the third stage of screening. In the final stage, the full texts were evaluated, and two more criteria were added: (i) participants were diagnosed with psychotic disorder or at least had been reported to have a high level of symptoms on at least one relevant measure for screening psychotic symptoms and (ii) there were at least two assessment points: pre- and post-treatment with at least 1 week in between. Follow-up assessment point was not compulsory for study inclusion, but in order to be considered as a follow-up point, there had to be at least 1 month between post-treatment and follow-up.

After the researchers had rated all the studies independently based on the title and abstract, a comparison between two independent ratings (VG vs. HL-S and BM) was made and a consensus on the studies to be included in the final stage of the screening for full-texts was reached. When the full text was not available in the searched databases, the researchers contacted the corresponding author. In case of no response in 2 weeks, the study was counted as missing. After the researchers had rated the full texts independently, a comparison of the ratings was made again, and a consensus on the studies to be included in the systematic review was reached. For a more detailed description of the study selection process see Vousoura et al. (22).

### Quality Assessment

The methodological quality of the included studies was evaluated by the Mixed Methods Appraisal Tool (MMAT) (27) for qualitative, quantitative, and mixed methods. Studies in the present review belong in the category of quantitative designs (RCTs and non-randomized trials) and were evaluated according to the relevant criteria. For RCTs, evaluation criteria involved randomization process, comparisons of groups at baseline, completion of the outcome data (by most of the participants), blinding of the assessors to the provided intervention, and adherence of the participants to the assigned intervention. With respect to non-randomized trials, criteria assessed whether the participants were representative of the target population, if measurements were appropriate regarding the outcome and intervention, completion of the outcome data (by most participants), consideration of confounding bias, and whether the intervention was administered as intended. Given that there is no strict value for acceptable complete outcome data, recommendation of MMAT (27) refers to the dropout/withdrawal rates that range between 5, 20, and 30% for a follow-up longer than 1 year. In this study, outcome data for both randomized and non-randomized trials was considered complete if the dropout rate was a maximum of 20% at post-treatment and 30% for a follow-up of more than 1 year. For every included trial, each criterion was rated as “yes,” “unclear,” or “no.”

## Results

### Study Selection

The search strategy identified 1,535 publications from the two databases. After removing duplicates, 1,449 publications were included in the screening process. From these, 328 were selected to be included in the final stage of screening. However, in total 31 papers were not available, so 297 full-text articles were assessed for eligibility. A total of 37 publications were selected, and three relevant articles were identified outside the systematic search (e.g., from references of selected papers), so in total 40 publications were included in this study. The PRISMA flow diagram detailing the number of studies retained for analysis according to the screening steps is presented in Figure 1.

### Study Design and Population

A total of 25 studies (40 publications) were included in the systematic review based on the defined eligibility criteria. Except for one study (28), all included studies (29–67) had control groups [e.g., TAU, waiting list (WL), no psychosocial treatment (NT), non-structured group intervention (NS)], and 19 studies were RCTs (29, 30, 32–34, 38–44, 46, 49–59, 63–67). In addition, all except for one study (40–44) reported the target intervention to be effective in treating psychotic patients. Therefore, we report results on the effectiveness of the treatments in comparison to the control treatments except for the one study (28) without a control group, where we report statistically significant pre-post differences.

### Interventions

Based on which type of psychological intervention was the target condition in the study, included studies were divided into three categories: (1) cognitive therapy (CT), cognitive-behavioral therapy (CBT), behavioral therapy (BT); (2) cognitive remediation therapy (CRT); (3) other psychological interventions. Detailed descriptions of the psychological interventions in the three defined categories are presented in Supplementary File 2. In addition to psychological interventions, most patients in all included studies were prescribed and/or received antipsychotic medication; three studies also reported the use of other psychotropic medication (e.g., antidepressants, mood stabilizers, benzodiazepines) (29–32).

#### Cognitive Therapy, Cognitive-Behavioral Therapy, Behavioral Therapy

Cognitive therapies focus on changing unhelpful cognitive distortions (e.g., thoughts, beliefs) and behaviors (BT, CBT), improving emotion regulation, and developing personal coping strategies targeting current problems. Therefore, frequently encountered topics among cognitive and behavioral interventions for psychotic disorder involve processing of and adaptation to illness (e.g., persistent symptoms), illness management (e.g., functional recovery), relapse prevention, and treatment motivation. Interventions follow standardized manuals with areas of psychological work and goals that are determined in advance. In addition, psychosocial programs in this category often involve psychoeducation addressing various topics (e.g., confidence, identity, stigma, substance use) (for details see Supplementary File 2).

Ten included studies (18 publications) belong in this category. Five studies had sample sizes of more than 50 participants (33–45) and two studies (28, 46) had less than 20 participants. Regarding the number of sessions, six studies involved designs with more than 16 sessions (28, 33–37, 40–46). The length of treatment in the included studies in this group involved periods from 4 to 19 weeks (32, 38, 39, 46–48) to more than 7 months (33–37, 40–44). Eight studies in this category reported results at follow-up (32–46, 48). An overview of the included studies with the main study characteristics and outcomes in this category is presented in Table 2.

**TABLE 2:** Studies concerning psychological interventions based on cognitive, cognitive-behavioral, or behavioral therapies for adolescents and young adults with psychosis.

Study	Patients N	Age (range, mean)	Control treatment	Diagnostics (assessment tool)	Frequency and number of sessions	Length of treatment	Outcome measurement instruments	Outcome, results (treatment vs. control)			Follow-up, results
								Symptoms	Functioning	Other	
**SCIT (CBT group)**											
Bartholomeusz et al. (28)	12	16–26; mean 21.6	–	DSM-IV-TR (SCID)	2 ×week; 20	10 weeks	**Symptoms:** BPRS, SANS, CESD; F**unctioning:** SOFAS, GFS: role and social, DANVA, hinting task, PST, IPSAQ	**Negative:** n/s; **Psychiatric symptoms (BPRS total):** n/s; **Depressive:** n/s	**Global functioning: Global functioning Role:** *p* = 0.03; **Global functioning social:** *p* > 0.001; **Social and occupational functioning:** n/s; **Cognitive functioning:** Emotion recognition, faces (low-intensity): *p* = 0.04; Emotion recognition, faces (high-intensity): n/s; Emotion recognition, paralanguage (low-intensity): n/s; Emotion recognition, paralanguage (high-intensity): n/s; Attributional bias: n/s; **Metacognitions:** Theory of mind: n/s	–	–
**CBTpA and FIpA (CBT individual and family)**											
Browning et al. (47)	30	14–17; mean 16.9, CBTpA mean 16.9, FIpA mean 16.9, SC mean 16.9	TAU	ICD-10	Up to 2 × week (total 5 h); CBTpA 10, FIpA 5	4–10 weeks	**Symptoms:** BPRS; F**unctioning:** C-GAS; **Other:** self-report	**Psychiatric symptoms (BPRS total):** n/s	**Global functioning:** n/s	**Length of hospitalization:** n/s; T**reatment satisfaction:** CBTpA = FIpA > TAU, *p* = n/a	–
**RPT (CBT)**											
Gleeson et al. (33, 34) (RCT)	81	15–25; mean 20.1, RPT mean 20.1, TAU mean 20.2	TAU	DSM-IV (SCID)	1 × 2 weeks, RPT mean 8.51	7 months	**Symptoms:** MADRS, BPRS, SANS; F**unctioning:** PAS, SOFAS, WHOQOL-BREF;	**Negative (alogia):** TAU > RPT, *p* < 0.001; **Negative (other):** n/s; **Psychiatric symptoms (BPRS**	**Premorbid adjustment:** n/s; **Social and occupational functioning:** n/s; **Quality of life:** n/s	**Relapse:** RPT > TAU, *p* = 0.042; **Medication adherence:** n/s; P**rescription of**	**12 months: Symptoms:** n/s; **Functioning:** n/s; **Other:** Relapse: RPT > TAU, *p* = 0.039;
							**Other:** MARS, WHO ASSIST, AUDIT, SDS, medical records, phone contact	**total):** n/s; **Depressive:** n/s		**medication:** n/s; S**ubstance use:** n/s	Medication: n/s; Substance use: n/s; **18–24 months: Symptoms:** Negative (alogia): TAU > RPT, *p* = 0.002–0.041; Negative (other): n/s; **Functioning:** n/s; **Other:** Relapse: n/s; **30 months: Symptoms:** Negative (alogia): TAU > RPT, *p* = 0.002; Negative (attention): TAU > RPT, *p* = 0.001; Negative (overall): TAU > RPT, *p* = 0.018; **Functioning:** Social and occupational functioning: TAU > RPT, *p* = 0.043; **Other:** Medication adherence: RPT > TAU, *p* = 0.045
**HYPE (CAT)**											
Gleeson et al. (46) (RCT)	16	15–25; mean 18.4, HYPE mean 18.6, TAU mean 18.3	TAU	DSM-IV-TR, psychosis and borderline (SCID)	1 ×week; 16	16 weeks	**Symptoms:** MADRS, BPRS, SANS; F**unctioning:** SOFAS; **Other: AIAQ, OAS-M,** ASSIST, AUDIT, SDS, MARS, medical records	**Positive:** HYPE > TAU, *p* = n/a; **Negative:** HYPE > TAU, *p* = n/a; **Psychiatric symptoms (BPRS total):** n/s; **Affective flattening:** n/s; **Alogia:** n/s;	**Social and occupational functioning:** HYPE > TAU, *p* = n/a	**Anger:** n/s; **Irritability:** HYPE > TAU, *p* = n/a; **Assault:** n/s; **Alcohol use:** TAU > HYPE, *p* = n/a; **Medication adherence:** HYPE > TAU, *p* = n/a	**6 months: Symptoms:** Negative: n/s; Positive: n/s; Psychiatric symptoms (BPRS total): HYPE > TAU, *p* = n/a; Depression: n/s; **Functioning:** Social and
								**Avolition:** n/s; **Anhedonia:** HYPE > TAU, *p* = n/a; **Attention:** n/s; **Depression:** HYPE > TAU, *p* = n/a			occupational functioning: HYPE > TAU, *p* = n/a; **Other:** Anger: HYPE > TAU, *p* = n/a; Irritability: HYPE > TAU, *p* = n/a; Assault: HYPE > TAU, *p* = n/a; Alcohol use: TAU > HYPE, *p* = n/a
**COPE (CT)**											
Jackson et al. (35–37)	80	16–30; COPE mean 21.39, TAU mean 21.93, NT mean 20.95	TAU, NT	DSM-III-R (RPMIP)	1 × week-1 × 2weeks, mean 18.0	12 months	**Symptoms:** BPRS, SANS, BDI-13, GSI of SCL-90-R; F**unctioning:** QLS; **Other:** EM, I/SO, medical records	**Negative symptoms:** TAU = COPE > NT, *p* > 0.05; **Psychiatric symptoms (BPRS total):** n/s; **General symptoms:** n/s; **Depressive:** TAU > COPE = NT, *p* < 0.05	**Quality of life:** TAU = COPE > NT, *p* > 0.05	**Understanding the illness:** TAU = COPE > NT, *p* < 0.05; **Adaptation to illness:** TAU < COPE > NT, *p* < 0.05	**12 months: Symptoms:** Psychiatric symptoms (BPRS total): n/s, Negative: n/s, Depression: n/s, General symptoms (GSI). n/s; **Functioning:** Quality of life: n/s; **Other:** Understanding the illness: n/s, Adaptation to illness: COPE > TAU, *p* = 0.008, Hospitalization: n/s; **4 years:** All outcome measures: n/s, Hospitalization: n/s
**ACE (CBT)**											
Jackson et al. (38) and Allott et al. (39) (RCT)	62	15–25; ACE mean 22.13, BE mean 22.45	BE	DSM-IV-TR (SCID)	Max. 20; mean 9.0; BE mean 7.2	12–14 weeks	**Symptoms:** BPRS, SANS; F**unctioning:** SOFAS; **Other:** CTRS	**Positive:** n/s; **Negative:** n/s	**Social and occupational functioning:** n/s	**Treatment adherence:** ACE > BE, *p* > 0.01	**12 months: Symptoms:** Negative = n/s; Positive: n/s;
											**Functioning:** Social and occupational functioning: n/s; **Other:** Hospitalization**:** n/s
**IPFI (family BT)**											
Lenior et al. (40), Linszen et al. (41–43), and Nugter et al. (44) (RCT)	76	15–26	TAU	DSM-III-R	Max. 18, mean 17	12 months	**Other:** BPRS-E, CFI, FMSS, LCS, medical records/clinical judgment	–	–	**Relapse:** n/s; **Family expressed emotions EE:** n/s; **Family criticism/ dissatisfaction CRIT:** n/s; **Family emotional overinvolvement EOI:** n/s	**5 years: Relapse rate:** n/s
**MAPS (CBT + family intervention)**											
Morrison et al. (45)	61	14–18; mean 16.3	MD	ICD-10	CBT 1 × week, max. 26 + family intervention 1 × month, max. 6	6 months	**Symptoms:** PANNS, PEQ; **Other:** QPR	**PANNS total:** n/s; **Positive:** n/s; **Negative:** n/s; **Psychotic experiences/ Disorganized:** n/s; **Psychotic experiences/ Excitement:** n/s; **Emotional distress:** n/s	–	**Subjective recovery:** n/s	**12 months: Symptoms:** PANNS total: n/s; Positive: MAPS < MAPS + MD; Negative: n/s; **Other:** Subjective recovery: MAPS < MAPS + MD > MD < MAPS
**CBT (group)**											
Newton et al. (48)	22	15–21; mean 17.0	WL	Distressing auditory hallucinations, no diagnosis	1 × week; 7	7 weeks	**Symptoms:** PSYRATS, PANSS, BDI, BAI; **Functioning:** CSQ, Activities Scale; **Other:** RSE, BIS, BAVQ	**PANNS total:** n/s; **Auditory hallucinations:** n/s; **Depression:** n/s; **Anxiety:** n/s	**Coping:** n/s	**Self-esteem:** n/s; **Control over voices:** CBT > WL, *p* = 0.04; **Power over voices:** CBT > WL, *p* = 0.04	**3 months:** Results remain
**GRIP (CBT)**											
Penn et al. (32) (RCT)	46	GRIP mean 23.5, TAU mean 21.0	TAU	DSM-IV (SCID)	1 ×week; max 36, mean 19	9 weeks	**Symptoms:** PANSS, CDSS; **Functioning:** QLS, RFS, MCAS, SSPA; **Other:** SPWB, MSPSS, AUS, DUS, BEMIB, self-report	**Positive:** n/s; **Negative:** n/s; **General psychopathology:** n/s; **PANSS total;** n/s; **Depressive:** n/s	**Work functioning:** n/s; **Quality of life:** n/s; **Social functioning:** n/s	**Psychological wellbeing:** n/s; **Perceived social support:** n/s; **Alcohol use:** n/s; **Attitude toward medication:** n/s; **Hospitalization:** n/s	**3 months: Symptoms:** Positive: n/s; Negative: n/s; General psychopathology: n/s; PANSS total: n/s; Depressive: n/s; **Functioning:** Work functioning: GRIP > TAU, *p* = n/a; Quality of life: n/s; Social functioning: n/s; **Other:** Psychological wellbeing: n/s; Perceived social support: TAU > GRIP, *p* = n/a; Alcohol use: n/s; Attitude toward medication: n/s; Hospitalization: n/s

*^a^The conclusion is based on the number of outcome comparison results presented. If more than 50% of the results are favoring one treatment condition, it is categorized as superior, if less than 50% it is equal or superior, if mixed results or no difference then it is equal.*

*^b^Treatments: ACE, CBT for early psychosis; BE, befriending; BT, behavioral therapy; CAT, Cognitive analytic therapy; CBT, cognitive behavioral therapy; CBTpA, CBT for adolescent patients with psychosis; COPE, cognitively oriented psychotherapy for early psychosis; FIpA, Family intervention for adolescent patients with psychosis; HYPE, Helping Young People Early; IPFI, TAU plus behavioral family intervention; MAPS, Managing Adolescent first episode Psychosis; MD, medication; NT, no psychosocial treatment; RPT, relapse prevention therapy; SCIT, social cognition and interaction training; TAU, treatment as usual; Diagnostic Assessment: DSM, Diagnostic and Statistical Manual of Mental Disorders; ICD, International Statistical Classification of Diseases and Related Health Problems; RPMIP, Royal Park Multidiagnostic Instrument for Psychosis; SCID, Structured clinical interview for DSM; Outcome Measurement Instruments: AISQ, Anger; Irritability and Assault Questionnaire; AUDIT, Alcohol Use Disorders Identification Test; AUS, Alcohol Use Scale; BAI, Beck Anxiety Inventory; BAVQ, Beliefs about Voices Questionnaire; BDI, Beck Depression Inventory; BEMIB, Brief Evaluation of Medication Influence and Beliefs; BIS, Birchwood insight scale; BPRS, Brief Psychiatric Rating Scale; CDSS, Calgary Depression Scale for Schizophrenia; CESD, Centre for Epidemiological Studies Depression Scale; CFI, Camberwell Family Interview; CGAF, Children‘s Global Assessment Scale; CSQ, Coping Strategies Questionnaire; CTRS, Cognitive Therapy Rating Scale; DANVA, Diagnostic Analysis of Non-verbal Accuracy-2: emotion perception hinting task; DAST, Drug Abuse Screening Test; DUS, Drug Use Scale; EM, Explanatory Model Scale = understanding beliefs about one’s illness; FESFS, First Episode Social Functioning Scale; FMSS, Five Minute Speech Sample; expressed emotion (EE): critical comments (CRIT), hostility, emotional over-involvement (EOI); GFS, Global Functioning Scales: role and social; GSI, General Symptom Index of SCL-90-R, HADS, Hospital Anxiety and Depression Scale; IPSAQ, Internal, Personal and Situational Attributions Questionnaire = attributional bias; I/SO, Integration/Sealing over = adaptation to illness; LCS, Life Chart Schedule = symptoms, treatment, social conditions; MADRS, Montgomery-Asberg Depression Rating Scale; MARS, Medication Adherence Rating Scale; MCAS, Multnomah Community Ability Scale; MSPSS, Multidimensional Scale of Perceived Social Support; OAS-M, Overt Aggression Scale-Modified for outpatients = suicidality and aggression; PANSS, Positive and Negative Symptoms Scale; PAS, Premorbid Adjustment Scale; PST, picture sequencing task = theory of mind (ToM); PSYRATS, Auditory Hallucinations Rating Scale; QLS, Quality of Life Scale (QLS); RFS, Role Functioning Scale; RSE, Rosenberg Self-Esteem Scale; SANS, Scale for the Assessment of Negative Symptoms; SDS, Substance Dependence Scale; SOFAS, Social and Occupational Functioning Assessment Scale; SPWB, Scales of Psychological Wellbeing; SSPA, Social Skills Performance Assessment; WHO ASSIST, Alcohol, Smoking, and Substance Involvement Screening Test; WHOQOL-BREF, World Health Organization Quality of Life Assessment.*

#### Cognitive Remediation Therapy

Cognitive remediation therapy mainly targets neurocognitive dysfunctions (e.g., working memory, attention, cognitive processing and flexibility), as well as metacognitive thinking (e.g., self-/illness awareness, insight, theory of mind) by using behavioral strategies to improve the targeted cognitive abilities and social functioning (for details of the interventions included in this category, see Supplementary File 2).

Nine included studies (13 publications) were designated under this category. Five of these studies had a sample size of fewer than 50 participants (49–56), three studies involved more than 50 participants (29, 30, 57, 58), and one study had more than 100 participants (59). With respect to the number of sessions, two studies had designs with up to 16 sessions (53–55, 57), and four studies provided more than 40 h of therapy (29, 30, 49, 56, 58). Treatment was delivered over 2 months or less in three studies (29, 30, 50, 53–55) from 3 to 6 months in five studies (51, 52, 56–59) or in a 12-month period in one study (49). Results at follow-up were reported by five studies in this group (51–56, 58, 59). An overview of the study characteristics and outcomes in this category is presented in Table 3.

**TABLE 3:** Studies concerning psychological interventions based on cognitive remediation for adolescents and young adults with psychosis.

Study	Patients N	Age (range, mean)	Control treatment	Diagnostics (assessment tool)	Frequency and number of sessions	Length of treatment	Outcome measurement instruments	Outcome, results (treatment vs. control)			Follow-up
								Symptoms	Functioning	Other	
**Computer CRT**											
Corbera et al. (49) (RCT)	45 (total *n* = 112)	Mean 22.2	CS	DSM-IV (SCID)	Max. 100 h, mean 42.08	12 months	**Symptoms:** PANNS; F**unctioning:** WAIS-III, UPSA-B	**Positive:** CRT > CS, *p* = 0.031; **Negative:** n/s; **Hostility:** n/s; **Emotional discomfort:** n/s	**Cognitive functioning:** Letter-number-sequencing: n/s, Digit span: CRT > CS, *p* = 0.004; **Adaptive functioning:** n/s	–	–
**iPadCT**											
Dang et al. (50) (RCT)	20 (males)	iPadCT mean 25.4, CG mean 25.0	CG	DSM-IV	5 × week	4 weeks	**Functioning:** N-back task	–	**Cognitive functioning:** Accuracy rate (2-back): iPadCT > CG, *p* > 0.01; Reaction times (0, 1, and 2-back): iPadCT > CG, *p* > 0.05	–	–
**AT**											
Fisher et al. (29) and Puig et al. (30) (RCT)	86	16–30; AT mean 21.7, CG mean 20.7	CG	DSM-IV (SCID)	5 × week, 40	8 weeks	**Symptoms:** PANNS; F**unctioning:** Global cognition (average z-score from all cognitive measures), TMT, WMS-III, HVLT-R, BVMT-R, D-KEFS, Strauss Carpenter Outcome Scale, Global functioning Role and Social (clinician rating); Other: Treatment adherence (%), enjoyment	**Positive:** n/s; **Negative:** n/s; **General psychopathology:** n/s; **PANNS total:** n/s	**Cognitive functioning:** Global cognition: AT > CG, *p* < 0.01; Speed of processing: n/s; Working memory: n/s; Verbal learning: n/s; Verbal memory: AT > CG, *p* < 0.01; Visual learning: n/s; Visual memory: n/s; Problem solving: AT > CG, *p* = 0.03; **Global functioning:** General functioning (Strauss Carpenter): n/s;	**Treatment adherence:** n/s; **Enjoyment:** n/s	–
									Global functioning Role: n/s, Global functioning Social: n/s		
**RECOS/REMAu/MBCT**											
Lalova et al. (57) (RCT)	63	18–25; mean 22.5 (REMAu), 22.6 (RECOS, 22.7 (MBCT)	3 different CR: RECOS, REMAu, MBCT	DSM-IV (PANNS)	1 × week; 12	3 months	**Functioning:** Stroop, WAIS-III, TMT, CVLT-II, WCST, Rey figure, TEMPAu, TSCS-II, RSCS, ToM, SSTICS, CDiS, MAAS; **Other:** SUMD	–	**Cognitive functioning:** Memory: n/s; Executive functions: RECOS > REMAu = MBCT, *p* > 0.01; Attention and processing speed: n/s; Autobiographical memory REMAu > RECOS = MBCT, *p* > 0.001; **Metacognition:** ToM: MBCT > RECOS = REMAu, *p* < 0.05; Self-concept: MBCT > RECOS = REMAu, *p* < 0.05; Satisfaction: MBCT > RECOS = REMAu, *p* < 0.05; Subjective complaints: RECOS > REMAu = MBCT, *p* < 0.05; mindful attention awareness: MBCT > RECOS = REMAu, *p* < 0.001	**Symptomatic awareness:** REMAu > RECOS = MBCT, *p* < 0.05; **Symptomatic attribution:** MBCT > RECOS = REMAu, *p* < 0.001	–
**CRT**											
Østergaard Christensen et al. (59) (RCT)	117	CRT mean 25.0, TAU mean 24.9	TAU	ICD (PSE)	2 × week CRT and 1 × 2 weeks competence dialog, 38, mean 28.7	16 weeks	**Symptoms:** PANNS; F**unctioning:** UPSA-B, MCCB, TMT, CPT-PI, WMS-III, HVLT-R, NAB Mazes, MSCEIT, DART; **Other:** RSE	**Positive:** n/s; **Negative:** n/s, **General psychopathology:** CRT > TAU, *p* < 0.05	**Cognitive functioning:** Speed of processing: n/s; Attention/vigilance; n/s; Working memory: n/s; Verbal learning: CRT > TAU,	**Self-esteem:** CRT > TAU, *p* > 0.05	**300 days:** Symptoms: General psychopathology: n/s; Positive symptoms: CRT > TAU, *p* = 0.04; Cognition: Working memory:
									*p* = 0.46; Visual learning: n/s; Reasoning and problem solving: n/s; Social cognition: n/s; MCCB composite: n/s; **Functional capacity:** n/s		CRT > TAU, *p* > 0.05; Verbal learning: CRT > TAU, *p* < 0.05; HVLT-R recall: CRT > TAU, *p* > 0.01; Other: Self-esteem: n/s; Medication compliance: n/s
**CRT**											
Puig et al. (58) (RCT)	50	12–18; CRT mean 16.7, TAU mean 16.8	TAU	DSM-IV-TR	2 × week, 40	20 weeks	**Symptoms:** PANNS, CDS; F**unctioning:** WMS-III, RAVLT, WISC-IV/WAIS-III, TMT, WCST, COWAT, LSP, VABS, C-GAS; O**ther:** RSE, CBI	**PANNS total:** n/s; **Depression:** n/s	**Global functioning (VABS):** CRT > TAU, *p* = 0.31; **Global functioning (C-GAS):** n/s; **Life skills:** CRT > TAU, *p* = 0.039; **Cognitive functioning:** Verbal memory: CRT > TAU, *p* = 0.003; Visual memory: n/s; Working memory: CRT > TAU, *p* = 0.041; Processing speed: n/s; Executive functions: CRT > TAU, *p* = 0.019; Cognitive composite score: CRT > TAU, *p* = 0.009	**Self-esteem:** n/s; **Cargiver burden:** CRT > TAU, *p* = 0.011	**3 months** (only CRT): **Functioning:** Cognitive functioning: Results remain; Global functioning: n/s
**Computer CRT**											
Ueland and Rund (51, 52) (RCT)	26	12–18; CRT mean 15.2, PE mean 15.4	PE	DSM-IV (SCID)	30 h	6 months	**Symptoms:** BPRS; F**unctioning:** BMT, SPAN, CPT, WCST, TMT, GAS, CBCL	**Positive:** n/s; **Negative:** n/s; **Psychiatric symptoms** (BPRS total): n/s	**Global functioning:** n/s; **Behavioral functioning:** n/s;	–	**12 months: Symptoms:** n/s; **Functioning:** Early visual information processing:
									**Cognitive functioning:** Attention: n/s; Memory: n/s, Executive functions: n/s		CRT > PE; All other: n/s
**CACR**											
Urben et al. (53), Pihet et al. (54), and Holzer et al. (55) (RCT)	32 (21 psychosis, 11 high risk)	13–18; CACR mean 15.4, CG mean 15.7	CG	DSM-IV (DIGS, also SIPS + SOPS for high risk)	2 × week, 16	8 weeks	**Symptoms:** PANNS; F**unctioning:** RBANS, SOFAS, HoNOSCA; O**ther:** Treatment engagement (motivation and engagement, 5-point scales)	**Positive symptoms:** n/s; **Negative symptoms: n/s; General Psychopathology:** n/s; **PANSS total:** n/s; **Health (HoNOSCA):** n/s	**Social and occupational functioning:** n/s; **Cognitive functioning:** Neuropsychological status (RBANS total): n/s; Immediate memory: n/s; Visuospatial/ constructional: CACR > CG, *p* > 0.05; Language: n/s; Attention: n/s; Delayed memory: n/s	**Treatment engagement:** n/s	**6 months** (from baseline): **Symptoms:** n/s; **Functioning:** Cognitive functioning: n/s
**CRT**											
Wykes et al. (56) (RCT)	40	14–22; mean 18.2, CRT mean 18.8, TAU mean 17.5	TAU	DSM-IV	3 × week, 40	3 months	**Symptoms:** BPRS; F**unctioning:** WCST, WAIS-R, SET, QoL, SBS; O**ther:** RSE	**Psychiatric symptoms** (BPRS total): n/s	**Social functioning:** n/s; **Quality of life:** n/s; **Cognitive functioning:** Cognitive flexibility: CRT > TAU, *p* = 0.04; Memory: n/s; Planning: n/s	**Self-esteem**: n/s	**3 months: Symptoms:** n/s; **Functioning:** Cognitive functioning: results remain; Social functioning: n/s; **Other:** Self-esteem: n/s

*^a^The conclusion is based on the number of outcome comparison results presented. If more than 50% of the results are favoring one treatment condition, it is categorized as superior, if less than 50% it is equal or superior, if mixed results or no difference then it is equal.*

*^b^Treatments: AT, computerized auditory training; CACR, computer assisted cognitive remediation; CG, computer games; CRT, cognitive remediation therapy; CS, computer skills training; iPadCT, iPad assisted cognitive training; MBCT, Mindfulness-based Cognitive Therapy; PE, psychoeducation; REMAu, The Autobiographical Reminiscence Therapy; RECOS, The Cognitive Remediation program for patients with Schizophrenia; TAU, treatment as usual; Diagnostic Assessment: DIGS, Diagnostic Interview for Genetic Studies; DSM, Diagnostic and Statistical Manual of Mental Disorders; PSE, Present state Examination interview; SCID, Structured clinical interview for DSM; SIPS, Structured Interview for Prodromal Symptoms; SOPS, Scale of Prodromal Symptoms; Outcome Measurement Instruments: BMT, Backward masking test; BPRS, Brief psychiatric rating scale; BVMT-R, Brief Visuospatial Memory Test-Revised; CBCL, Child Behavior Check List; CBI, Caregiver Burden Inventory; CDS, Calgary Depression Scale; CDiS, The Cognitive Difficulties Scales; C-GAS, The Children’s Global Assessment Scale; COWAT, Controlled Oral Word Association Test; CPT, Degraded stimulus continuous performance test; CVLT, California Verbal Learning Test; DART, Danish Adult Reading Test; D-KEFS, Delis-Kaplan Executive Function System; GAS, Global Assessment Scale; HoNOSCA, Health of Nation Outcome Scale for Children and Adolescents; HVLT-R, Hopkins verbal learning test-revised; LSP, Life Skills Profile; MAAS, The Mindful Attention Awareness Scale; MCCB, MATRICS Consensus Cognitive Battery; MSCEIT, Mayer–Salovey–Caruso Emotional Intelligence Test; NAB Mazes, Neuropsychological Assessment Battery; PANSS, Positive and Negative Syndrome Scale; QoL, Quality of Life Scale; RAVLT, Rey Auditory Verbal Learning Test; RBANS, Repeatable Battery for the Assessment of Neuropsychological Status; SBS, Social Behavior Schedule; SET, Six Elements Test; RSCS, Revised Self-Consciousness Scale; RSE, Rosenberg Self-Esteem Scale; SOFAS, Social and Occupational Functioning Assessment Scale; SPAN, Span of Apprehension Task; SSTICS, The Subjective Scale to Investigate Cognition in Schizophrenia; SUMD, Scale to assess Unawareness of Mental Disorder; TEMPAu, Test Episodique de Mémoire du Passé Autobiographique; TMT, Trail Making Test; ToM, Theory of mind; TSCS, The Tennessee Self Concept Scale; UPSA-B, University of California San Diego Performance Skills Assessment; VABS, The Vineland Adaptive Behavior Scales; WAIS, Wechsler Adult Intelligence Scale; WCST, Wisconsin Card Sorting Test; WMS, Wechsler Memory Scales.*

#### Other Psychological Interventions

The studies included in this category were a heterogeneous group, typically involving an integrative approach combining psychoeducation and/or individual treatment with family or group interventions (for details of the interventions included in this category, see Supplementary File 2).

This group of treatments comprised six included studies (nine publications). Except for one study (60–62), all studies in this category involved more than 50 participants; two studies had sample sizes of more than 100 participants (31, 63). In the terms of the number of sessions, almost all included studies in this group involved designs with 9–16 sessions; one study provided a significantly higher number of sessions (mean 184.4) than the other studies over the period of 1 year (31). Two studies had a treatment period of 3 months or less (64, 65). Half of the studies in this category delivered treatment in the period between nine and 12 months (31, 63, 66, 67), and one study had a treatment period up until two years (60–62). Two studies reported results at follow-up (65–67). An overview of the included studies in this category is presented in Table 4.

**TABLE 4:** Studies concerning other psychological treatments for adolescents and young adults with psychotic disorders.

Study	Patients N	Age (range, mean)	Control treatment	Diagnostics (assessment tool)	Frequency and number of sessions	Length of treatment	Outcome measurement instruments	Outcome, results (treatment vs. control)			Follow-up, results
								Symptoms	Functioning	Other	
**PGI**											
Calvo et al. (66, 67) (RCT)	55	14–18; PGI mean 16.4, NS mean 16.5	NS	DSM-IV (K-SADS-PL)	1 × 15 days, 15 (3 ind + 12 group) sessions	9 months	**Symptoms:** PANSS; F**unctioning:** C-GAS; O**ther:** questionnaire, FES	**Positive:** n/s; N**egative:** PGI > NS, *p* = 0.039; **PANSS total:** n/s	**Global functioning:** n/s	**Hospitalization:** n/s; **days in hospital:** n/s; **visits to emergency:** PGI > NS, *p* = 0.039; **using pharmacological treatment:** n/s; f**amily environment:** n/s	**2 years: Symptoms:** Differences in diagnosis: n/s; Negative: n/s; Positive: n/s; PANSS total score: n/s; **Functioning:** Global functioning: n/s; **Other:** Hospitalization: n/s; Days in hospital: n/s, Visits to emergency: PGI > NS, *p* = 0.019
**EEI**											
Chang et al. (63) (RCT)	160	15–25; mean 22.9 (sd 3.2), EEI mean 23 (3.0), TAU mean 22.8 (3.3)	TAU	DSM-IV (SCID-I)	16	12 months	**Symptoms:** PANSS, CDS; F**unctioning:** SOFAS, RFS, functioning status (SOFAS + RFS,%)**; Other:** Remission: PANSS (%); medical records	**Positive:** n/s; **Negative:** EEI > TAU, *p* = 0.013; **General psychopathology:** EEI > TAU, *p* = 0.01; **Depressive:** EEI > TAU, *p* = 0.008	**Functioning status:** EEI > TAU, *p* = 0.022; **Psychosocial functioning: SOFAS total:** EEI > TAU, *p* = 0.001; **RFS total:** EEI > TAU, *p* = 0.002; I**ndependent living skills:** EEI > TAU, *p* = 0.036; W**ork productivity:** EEI > TAU, *p* = 0.045; **Relationships of immediate networks:** EEI > TAU, *p* = 0.002; R**elationships of extended social networks:** EEI > TAU, *p* = 0.004	**Remission:** n/s; H**ospitalization:** n/s; D**ays in hospital:** n/s; D**efault in outpatient treatment:** EEI > TAU; *p* = 0.029, **Relapse:** n/s; **full time work:** n/s; U**sing pharmacological treatment:** n/s; T**reatment adherence:** n/s	–
**PST**											
Koren and Stepunina (64) (RCT)	56	15–17; mean 17.4	NT	ICD-10	1 × week, 12 sessions	3 months	**Symptoms:** PANSS; **functioning:** C-GAS; PedsQL	**Symptom severity:** n/s	**Global functioning:** n/s; **Quality of life (PedsQL total):** PST > NT, *p* > 0.05; **Social functioning:** PST > NT, *p* > 0.05; P**arent’s assessment for social functioning:** PST > NT, *p* < 0.01; **Parent’s assessment for** s**chool functioning:** PST > NT, *p* < 0.05	–	–
**EDIC**											
Lambert et al. (31)	225	12–29; EDIC mean 21.2 (sd 4.0), TAU mean 20.9 (sd. 4.2)	TAU	DSM-IV-TR (SCID-I and II)	EDIC: 3.5 × week, mean 184.4; TAU: mean 15.6	12 months	**Symptoms:** PANSS; F**unctioning:** GAF; **Other:** Remission:% of patients reporting remission in PANSS and GAF for ≥ 6 months; DUP: Royal Park Multidiagnostic Instrument for Psychosis Part I and II	**Positive:** EDIC > TAU, *p* = 0.014; **Negative:** n/s; **General psychopathology:** n/s; **PANSS total:** n/s	**Global functioning:** EDIC > TAU, *p* = 0.010	**Remission:** EDIC > TAU, *p* < 0.001; **DUP:** EDIC > TAU, *p* < 0.001	–
**IPEF**											
Rund (60) and Rund et al. (61, 62)	24	13–18; mean 16.0	TAU	DSM-III-R (SCID)	Inpatient: 1 × 2 weeks, outpatient:1 × 1–2 months	2 years (inpatient: months- 1 year, outpatient: until 2 years)	**Functioning:** GAS; **Other:** Relapse (5-point scale); CFI	–	**Global functioning:** n/s	**Relapse:** IPEF > TAU, *p* < 0.01; **Parental expressed emotions:** IPEF > TAU, *p* = n/a	–
**SGT**											
She et al. (65) (RCT)	60	16–18; mean 16.7	HG	DSM-IV	2 × week, 12 sessions	6 weeks	**Symptoms:** PANSS; O**ther:** SCCS	**Positive:** SGT > HG, *p* < 0.05; **Negative:** n/s, **General psychopathology:** SGT > HG, *p* < 0.001; **PANSS total:** SGT > HG, *p* < 0.001	–	**Self-consistency and congruence**: SGT > HG, *p* < 0.001	**3 months: Symptoms:** Positive: SGT > HG; Negative: n/s; General psychopathology: SGT > HG; PANSS total: SGT > HG; **Other:** Self-consistency and congruence: SGT > HG; **12 months:** n/s

*^a^The conclusion is based on the number of outcome comparison results presented. If more than 50% of the results are favoring one treatment condition, it is categorized as superior, if less than 50% it is equal or superior, if mixed results or no difference then it is equal.*

*^b^Treatments: EDIC, early detection plus integrated care; EEI, extended early intervention; HG, Handycraft Group; IPEF, Integrative psychoeducational family treatment; NS, non-structured group intervention; NT, no psychosocial treatment; PE, psychoeducation; PGI, psychoeducational group intervention; PST, group psychosocial therapy; SGT, Structural Group Therapy; TAU, treatment as usual; Diagnostic Assessment: DSM, Diagnostic and Statistical Manual of Mental Disorders; ICD, International Statistical Classification of Diseases and Related Health Problems; K-SADS-PL, The Schedule for Affective Disorders and Schizophrenia for School-Age Children –Present and Lifetime Version; SCID, Structured Clinical Interview for DSM; Outcome Measurement Instruments: CDS, Calgary Depression Scale; CFI, Camberwell Family Interview; C-GAS, Children’s Global Assessment Scale; DUP, Duration of psychosis; FES, Family Environment Scale; GAF, Global Assessment of Functioning Scale; GAS, Global Assessment Scale; PANSS, Positive and Negative syndrome Scale; PedsQL, Pediatrics Quality of Life Inventory; RFS, Role Functioning Scale; SCCS, Self-consistency and congruence scale; SOFAS, Social and Occupational Functioning Assessment Scale.*

### Outcomes of the Psychological Interventions

The results of the studies are reported within three categories of outcomes: (1) symptoms (positive, negative, general), (2) functioning (global functioning, social and occupational functioning, quality of life, cognitive functioning), and (3) other outcomes (relapse, hospitalization, medication, psychological and parental/familial variables). Results within each type of outcome will be presented for each category of psychological interventions. More specific details regarding the outcomes of included studies are presented in Tables 2–4.

#### Outcomes Focusing on Symptoms

The majority of the included studies investigated positive symptoms of psychosis (e.g., perceptual disturbances, delusions, hostility), negative symptoms of psychosis (e.g., anhedonia, avolition, blunted affect), and general symptoms (e.g., anxiety, depression, tension, poor attention).

Regarding *positive symptoms*, five studies from the CT/CBT/BT group reported results within this domain (32, 38, 39, 45, 46, 48). One study found significantly better results in the treatment group as opposed to TAU (46), while four reported no significant differences between treatment arms (32, 38, 39, 45, 48). However, the study that reported significantly better performance of the treatment group involved patients with a comorbid borderline personality disorder. With respect to the CRT group, five studies assessed positive symptoms (29, 30, 49, 51–55, 59), but only one reported a significant effect on positive symptoms particularly in the group of early-stage psychosis (25 years or younger) (49) compared to computer skills training. In the group of other psychological interventions, four studies investigated the effectiveness of treatment on positive symptoms (31, 63, 65–67), and two of them reported significant differences compared to the comparison treatment: early detection plus integrated care program (EDIC) (31) and structural group intervention (SGI) (65) were superior to TAU.

Concerning *negative symptoms*, seven studies out of ten in the CT/CBT/BT group reported outcomes under this domain (28, 32–39, 45, 46). Only Gleeson et al. (46) reported better outcomes for the treatment group compared to TAU concerning negative symptoms and anhedonia for the patients with a comorbid borderline personality disorder. Conversely, a significantly better outcome was reported for TAU regarding alogia in Gleeson et al. (33, 34). Four studies reported results of treatment interventions that were not significantly different to the comparison treatments (32, 35–39, 45). The non-RCT study (28) did not find a significant effect of the treatment. In the CRT group, five studies reported results on negative symptoms and found no statistically significant differences to the comparison treatments (29, 30, 49, 51–55, 59). Out of four studies in the group of other treatments that reported results regarding negative symptoms (31, 63, 65–67), two studies reported significantly better outcomes for the target intervention compared to control treatment: extended early intervention (EEI) was superior to TAU (63), and the psychoeducational group intervention (PGI) outperformed the NS (66, 67).

In terms of *general psychopathology*, the included studies reported results regarding depressive symptoms, anxiety, and general psychopathology status (e.g., PANSS total, BPRS) in the context of psychosis. In the CT/CBT/BT group, six studies investigated depressive symptoms (28, 32–37, 46, 48) and one study assessed anxiety (47) as treatment outcomes. Concerning depressive symptoms, only the Gleeson et al. study (46) found that the treatment group had significantly fewer positive symptoms than TAU group after the treatment. On the other hand, Jackson et al. (35–37) found that TAU had a significantly better outcome compared to the treatment group, even though the treatment group outperformed the third treatment arm with NT. Six studies in this group assessed outcomes regarding general symptoms status (32–37, 46–48) and reported no significant differences between treatment and control groups. Also, the non-RCT (28) found no treatment effect. Regarding the CRT group, out of all studies that reported results on depressive symptoms (58) and general symptoms status (29, 30, 49, 51–56, 58, 59), only Østergaard Christensen et al. (59) found significantly different outcome in general psychopathology for the cognitive remediation group as opposed to TAU. Wykes et al. (56) reported no evidence of (direct) effectiveness of CRT treatment on symptoms compared to TAU since the intervention did not target these domains specifically. However, improvements in cognition had beneficial effects on overall psychiatric symptoms. Within the group of other psychological interventions, only one studied the effectiveness on depressive symptoms63 and five studied general psychopathology as an outcome (31, 63–67). Extended early intervention (EEI) was more effective than TAU on depressive and overall psychopathology score (63). Additionally, structural group therapy (SGI) was significantly better than handicraft group (HGI) on general psychopathology status (65). The other studies reported no significant between-group differences.

#### Outcomes Focusing on Functioning

Twenty-two out of the 25 included studies explored different forms of functioning, e.g., global functioning, social and occupational functioning, quality of life, and cognitive functioning.

With respect to *global or social and occupational functioning and quality of life*, seven studies in the CT/CBT/BT group investigated this form of outcome (28, 32–34, 38, 39, 46–48). Significant differences between the treatment groups and no treatment were reported with respect to the quality of life (interpersonal and role functioning) (35–37) and social and occupational functioning (46). Penn et al. (32), Gleeson et al. (33, 34), Browning et al. (47), and Newton et al. (48) and did not find significant differences compared to control treatments and Bartholomeuz et al. (28) did not find a significant effect of the treatment in their uncontrolled study. Within the CRT group, out of six studies that reported results on this domain (29, 30, 51–56, 58, 59), only Puig et al. (58) found significantly better outcome for the treatment group regarding life skills. In the study by Wykes et al. (56), improvements in cognition had beneficial effects on social functioning, even if no direct effectiveness of CRT on social functioning was studied. Among the group of other psychological interventions, except for one article (65), all included studies explored global or social functioning, or quality of life. Significant improvements were reported in the treatment groups in comparison to TAU on global functioning for extended early intervention (EEI) (63) and early detection plus integrated care (EDIC) (31). Participants who attended early extended intervention (EEI) had significantly better functioning status on psychosocial functioning, independent living skills, work productivity, and relationships compared to participants receiving TAU (63). Additionally, patients receiving group psychosocial therapy (PST) had significantly better results on social functioning and quality of life, and also on parent’s assessments of social and school functioning compared to the group receiving NT (64). Rund (60), Rund et al. (61, 62), and Calvo et al. (66, 67) did not find any between-group effects.

Concerning *cognitive functioning*, only one uncontrolled study (28) in the CT/CBT/BT group investigated this domain. This study provided social cognition and interaction training and reported significant improvements in emotion recognition (for low-intensity facial expressions); changes in other cognitive domains (e.g., metacognition) were not statistically significant (28). One study in the CRT group (57) investigated metacognition [e.g., insight, autobiographical memory, self-concept, theory of mind (ToM)] by comparing three forms of psychotherapy: Cognitive Remediation program for patients with Schizophrenia (RECOS), Autobiographical Reminiscence Therapy (REMA), and Mindfulness-based Cognitive Therapy (MBCT). All metacognitive aspects improved regardless of the form of therapy, but RECOS outperformed two other treatments in one domain (subjective complaints) (57). Additionally, eight studies in this category reported significant differences in favor of the CRT treatment groups on cognitive functioning (29, 30, 49, 50, 53–59) and specifically on working memory (49, 58), verbal learning and memory (29, 30, 58, 59), executive functioning (57, 58), problem solving (29, 30) and accuracy rate and reaction times (50). Studies in the group of other psychological interventions had not investigated nor reported results regarding cognitive functioning.

#### Other Outcomes

Included studies also reported results that were related to other types of outcomes, such as relapse, hospitalization, medication, treatment adherence, psychological variables, or parental/family variables.

Regarding *relapse of psychosis*, two studies from the CT/CBT/BT group (33, 34, 40–44) and one study in the group of other treatments (60–62) explored this outcome. In comparison to TAU, significantly lower relapse rate was reported for the relapse prevention therapy (RPT) (33, 34) and the integrative psychoeducational family treatment (IPEF) (60–62). With respect to *hospitalization*, two studies from the CT/CBT/BT group (32, 47) and two studies from the group of other treatments (63, 66, 67) found no significant differences between treatment and control groups in hospitalizations. Concerning *adherence, prescription, or attitude toward medication*, two studies from the CT/CBT/BT (32–34, 46) and two from the group of other treatments (63, 66, 67) reported no significant differences to the control groups. Studies from the CRT group did not report results regarding relapse, hospitalization, or medication. With respect to *treatment adherence/satisfaction*, one study in the CT/CBT/BT group (29, 30) reported patients being more adherent to ACE than to the control treatment of befriending (BE), and another (47) found that patients receiving individual or family CBT were more satisfied than patients receiving TAU. In the two studies from the group of CRT (29, 30, 53–55) or one study in other treatments (63), no differences were found between the treatment and control groups.

In the group of CT/CBT/BT, three studies (32, 46, 48) reported outcomes on *psychological variables* such as self-esteem, psychological wellbeing, anger, or irritability. Only Gleeson et al. (46) found that a cognitive-analytic therapy program (HYPE) for the patients with a comorbid borderline personality disorder outperformed TAU on irritability, but not on the other psychological variables. In the CRT group, Østergaard Christensen et al. (59) found CRT to be superior to TAU for improving self-esteem. However, Wykes et al. (56) and Puig et al. (58) found no difference compared to TAU, nor did Fisher et al. (29) and Puig et al. (30) for enjoyment. For the group of other treatments, only one study reported psychological outcomes finding that patients receiving structural group therapy had better outcomes for self-consistency and congruence than patients in the handicraft group (HG) (65).

*Parental/familial variables* were investigated in the CT/CBT/BT group (40–44), CRT group (58), and the group of other treatments (60–62, 66, 67). In comparison to the control groups, significant improvements were reported with respect to the caregiver burden in the CRT group (58) and parental expressed emotions (EE) in the group of other treatments (60–62).

#### Outcomes at Follow-Up

Fifteen studies also reported results at follow-up, which involved periods between 3 months and 5 years (32–46, 48, 51–56, 58, 59, 65–67).

Included studies in the CT/CBT/BT group reported follow-up results at three to 6 months (32, 46, 48), 12 months (33–39, 46) or 2–5 years follow-up (33–37, 40–44). Gleeson et al. (46) reported that the treatment condition (HYPE) outperformed TAU on psychiatric symptoms (BPRS total), social and occupational functioning, anger, irritability, and assault, while TAU outperformed HYPE for alcohol use at 6-month follow-up. However, the study did not report on the statistical significance of the differences. Statistically significant improvements of the treatment group in comparison to TAU were reported with respect to adaptation to illness (35–37), and relapse and medication adherence (33, 34) at 12 months. TAU was superior on negative symptoms (alogia) and social and occupational functioning in the period between 18 and 30 months of follow-up (33, 34). In Morrison et al. (45), managing adolescent first episode psychosis—treatment program (MAPS) outperformed the group receiving only medication for subjective recovery, but the group receiving both outperformed MAPS.

Within the CRT group, studies reported follow-up results at 3–6 months (53–56, 58) and up to 12 months follow-up (51, 52, 59). Significant differences between the CRT intervention and TAU were found only in Ueland and Rund (51, 52) on early visual information processing at 12-month follow-up, and in Østergaard Christensen et al. (59) on positive symptoms, working memory, verbal learning and recall at 300 days follow-up.

In the group of other treatments, two studies reported outcomes at follow-up: She et al. (65) at three to 12 months and Calvo et al. (66, 67) at two years. Structural group intervention (SGI) outperformed handicraft group intervention (HGI) on positive symptoms, general psychopathology status, and self-consistency and congruence at 3-month follow-up (65). Similarly, psychoeducational group intervention (PGI) reported significantly better outcome on the visits to emergency compared to the NS at two-year follow-up (66, 67).

### Quality Assessment of the Included Studies

Out of twenty-five included original studies, nineteen studies were randomized control trials (RCTs), five were non-RCTs, and one was an uncontrolled study. With the exception of four studies (49, 50, 57, 64), all studies in the RCT group reported on the randomization process. Similarly, all studies involved groups that were comparable at baseline. The majority of RCTs reported outcome data that were completed by most of the participants (range of complete data: minimum of 80% at post-treatment and 70% for a follow-up of more than 1 year). Four studies in the RCT group did not meet this criterion (29, 46, 53, 58), and five studies were evaluated as “can’t tell” (32, 45, 56, 65, 66); specifically, in three studies, the criterion for completion of outcome data was met at the end of the treatment but not at follow-up (45, 56, 65), and in two studies many participants attended the assessments even if they did not continue receiving the treatment (32, 66). Regarding the blinding of the assessors to the provided interventions, twelve RCTs clearly reported and met this criterion. The remaining studies in this group either did not mention the blinding and were thus rated as “can’t tell” (50, 51, 57), reported blinding only for some assessments (56), acknowledged breaking the blind (45), or noted the absence of blinding due to the insufficient resources, as the trial was a pilot study (46). Most studies reported good adherence of participants to the assigned intervention, while six RCTs (29, 32, 46, 53, 58, 66) failed to meet this criterion.

With respect to the six included non-randomized trials (28, 31, 35–37, 47, 48, 60–62), criteria were completely met for the participants being representative of the target population, the use of appropriate measurements regarding the outcome and intervention (both of these being the inclusion criteria for the present review), and all studies reported administering the intervention as intended. Conversely, none of the included non-randomized trials accounted for the possible confounders in the analysis. Regarding the completion of the outcome data, three non-randomized trials met this criterion (47, 48, 60) while three studies had more than 20% drop-out rate (28, 31, 35); one study in the latter group had a small sample (*n* = 12), which impacted the approximate number of participants required to meet this criterion (*n* = 10) (28) (for more detailed description of the risk of bias and methodological quality assessment, see Supplementary File 3).

## Discussion

The aim of the systematic review was to present the research evidence from clinical trials on the effectiveness of psychological interventions for treating young people with psychotic disorders. We found 25 studies (in total 40 publications) meeting the inclusion criteria for this review. Most of the studied treatments have a cognitive or cognitive behavioral approach and also include family-related components and psychoeducation. The finding is in line with earlier studies on adult population (11, 68). The review focused mainly on the clinically relevant outcomes, such as symptom reduction or remission, hospitalization, and improvements in occupational, social, and cognitive functioning.

Regarding the effect of the psychological interventions on symptom reduction, only one (46) of the five studies assessing positive symptoms as an outcome from the CT/CBT/BT group, one (49) of five from the CRT group, and two (31, 65) of four from the group of other treatments reported that the target treatment significantly outperformed the control condition. For reduction in negative symptoms and general psychopathology the rates were even lower, as mostly no significant differences were found, and in one study (33, 34), TAU even outperformed the treatment condition. With respect to global or social and occupational functioning and quality of life, only two (35–37, 46) of seven found significant differences for CT/CBT/BT, one (58) of six for CRT and three (31, 63, 64) of six for other treatments. For cognitive functioning, only one non-controlled study (28) for CT/CBT/BT group and no studies for the other treatment group assessed this outcome. For the CRT group, all nine studies assessed cognitive functioning as an outcome and reported that CRT significantly outperformed the control treatment. Regarding relapse, one (33, 34) of two studies from the CT/CBT/BT group and one study (60–62) from the group of other treatments reported results favoring the treatment condition, whereas none of the four studies (32, 47, 63, 66, 67) assessing hospitalization as an outcome reported the treatment condition to outperform the control condition.

In the CT/CBT/BT group, the most promising treatment was cognitive-analytic therapy program (HYPE) (46), which outperformed TAU in most of the studied outcomes (positive and negative symptoms; anhedonia; depression; social and occupational functioning; irritability; medication adherence). However, as the number of participants was low (*n* = 16), significance testing, or calculation of effects sizes could not be performed. In addition, all patients had a comorbid borderline personality disorder, so the results should be interpreted with caution.

In the CRT group, the studies mainly showed that CRT resulted in significantly higher improvement of cognitive functioning than the control conditions. However, in terms of symptom reduction, only one study (49) showed CRT to reduce positive symptoms significantly more for CRT compared to computer skills training, and in one study (59) CRT outperformed TAU in reducing general psychopathology and increasing self-esteem.

In the heterogeneous group of other psychological interventions, in five out of six studies, the target intervention outperformed the control condition for most of the studied outcomes. The extended early intervention (EEI) (63) was significantly more effective than TAU in reducing symptoms (negative, depressive; general psychopathology) and improving functioning (global functioning, independent living skills, work productivity, relationships of intermediate and extended networks), and patients had fewer missed sessions from outpatient treatment. Furthermore, early detection plus integrated care (EDIC) (31) was superior to TAU in reducing positive symptoms and the duration of psychosis, increasing global functioning, and gaining remission; integrative psychoeducational family treatment (IPEF) (60–62) was more effective in preventing relapses and improving parental expressed emotions. Group psychosocial therapy (PST) (64) outperformed the group not receiving psychosocial treatment in improving social functioning and quality of life, and in school and social functioning as assessed by the parents. In addition, structural group therapy (SGT) (65) outperformed handicraft group in reducing positive symptoms, overall symptom severity and general psychopathology, and in improving self-consistency and congruence. However, as the group of other treatments was not unified, it is hard to draw firm conclusions of the effectiveness compared to the control treatments.

The main findings from this systematic review suggest that while psychological interventions have been found to be effective in reducing symptoms and improving functioning, psychotherapy does not typically outperform control conditions when it comes to symptom reduction, and the results from different studies do not seem to strongly favor any specific type of treatment. Similar findings were reported in a recent systematic review on psychological interventions for adults with schizophrenia or psychosis who received minimal or no antipsychotic medication (11), and in the meta-analysis by Datta et al. (21) for younger adolescents with psychotic disorders.

The review indicates that interventions with a bio-psycho-social integrative approach combining for example psychoeducation and family or group interventions might be more effective than control conditions in reducing symptoms and improving functioning. These results are in line with recent systematic reviews focusing on RCT studies for adolescents with psychotic disorders (21) and on psychosocial interventions aiming to improve social and occupational function in the early stages of psychosis (69), which concluded that “psychosocial interventions, particularly when provided as part of a multi-component intervention model and delivered in community-based settings are associated with significant improvements in social and occupational function.” It has been suggested that CBT would be the recommendable psychosocial treatment for adults with psychotic disorders (11, 70), but this outcome was not supported in our systematic review, which included young adults, since CBT outperformed the control treatments only in some of the outcome domains. Frawley et al. (69) also reported that interventions based on CRT significantly outperformed symptom-focused CBT interventions, while the largest gains were associated with multi-component interventions.

With regard to improvement of cognitive functioning, CRT relatively consistently outperformed control treatments. However, the implications of these findings for CRT as a general psychological approach for psychosis in young people are not clear, since studies of CRT mainly target cognitive domains, while CBT and other types of psychological interventions for psychosis focus more on symptoms and other types of functioning. Thus, the significant results of CRT typically regarded improvement in cognitive functioning, and a significant symptom reduction compared to the control treatment was found only in two studies. Conversely, apart from one study that did not have a control condition, studies with CT/CBT/BT or other types of psychological interventions did not investigate outcomes related to cognitive functioning. This means that in order to be able to compare if CRT is more effective in terms of cognitive improvement than other types of active psychological interventions, it is important that cognitive improvement would be systematically assessed as a treatment outcome across different interventions. And vice versa—in order to make conclusions if CRT is generally more effective than other types of psychological interventions for treating psychosis, a broader range of outcome measures should be added more systematically to studies assessing the efficacy of CRT.

There were remarkable differences in the designs of the studies. The duration of treatment, frequency, and number of sessions varied notably, and, furthermore, the control conditions provided varying degrees of support, which may result in differences in the perceived effects (6). Additionally, there was a wide variety of different types of outcomes assessed with different measures used in the studies, even if the results for each type of outcomes were considered “somewhat comparable” (MMAT), as validated instruments for the measurement were used. These findings are in line with the meta-analysis of Bighelli et al. (13) suggesting that considerable methodological improvement in studies on psychological treatments for schizophrenia would be crucial to have a higher confidence in the results. The evidence especially on CRT has increased in the last years, so in the near future, there might be stronger evidence on the effects for different types of treatment outcomes. The difficulty in summarizing the evidence and drawing conclusions from it is also partly due to the heterogeneity of the included interventions, even if belonging under the same treatment modality. Many clinics are developing their own slightly adjusted treatment programs instead of directly implementing treatment programs developed in other centers. Accordingly, there are many psychological interventions that are reported only in one study or studied only in one clinic, limiting our ability to draw conclusions on their effectiveness. In addition, in all included studies, participants were prescribed and/or received antipsychotic medication. However, only six studies considered this aspect as possibly affecting the results through: assessing medication use as a background factor (33, 34, 46), acknowledging medication effects as the response to the intervention (29), mentioning not controlling for the effects of medication to be a limitation (51), and implying that the positive findings were highly unlikely a result solely of medication (48). Only one study controlled for the medication and found no significant effects nor change in the results that could be attributable to this factor (56).

It is also notable, that the control treatments labeled as TAU varied considerably, in some cases being as intensive as the target treatment, being structured and provided by a specialist team for early psychosis, which might explain that there were not so many between-group differences found. The majority of studied psychological interventions were brief (less than 6 months), especially in the CT/CBT/BT and CRT groups, and follow-up was lacking in nearly half of the studies. Yet, it seems that the longer the follow-up is, the more likely it is that the possible between-group effects fade away. This is in line with what, e.g., Linszen et al. (43) and Harder et al. (71) hypothesized, that the best way to prevent poor outcomes in early recognized first episode schizophrenia would be sustained case management for at least 5 years to approach the critical period in which the severity of schizophrenia is established. However, in order to have more knowledge on the sustainability of the outcomes and be more convinced of the accuracy of the hypothesis, more studies with longer follow-ups are still needed.

### Strengths and Limitations

The primary strength of the systematic review is that as to our knowledge it is the first study to be looking into the full age range being the most essential period for the risk of developing a psychotic disorder (5). In addition, the study has reviewed the effectiveness of all types of psychological interventions, and that it has looked into a large variety of distinct outcomes.

The systematic review was conducted as a part of a larger study focusing on the full spectrum of psychiatric diagnoses. Thus, the strength of the study is that the systematic search was created by an interdisciplinary group of professionals in order to ensure that it was as inclusive as possible on the clinical outcome studies of psychological interventions for youth with mental disorders. In addition, the searches were strictly following the PICOS strategy. The task of the overall study was extensive resulting in a very inclusive and more diverse range of included studies compared to recent meta-analyses on specialist early intervention teams to treat recent onset psychosis (16) or psychological interventions for adolescents (21). Our approach to also include non-RCT studies in the review gives a broad overall picture on the treatments and studies conducted on treating young people with psychotic disorders.

In the terms of quality assessment, the majority of the included RCTs met the rating criteria (randomization, comparisons of groups at baseline, blinding). Relatively few studies in this group did not meet the criterion for a sufficient rate of completion of outcome data (29, 46, 53, 58), while a number of additional studies did not meet this criterion at follow-up (45, 56, 65). Similar evaluations were found for non-randomized trials for most of the criteria (representativeness of the participants for the target population, use of appropriate measurements for the outcome and intervention, and administration of the intervention as intended).

One of the principal limitations of the present review is the inclusion criteria, which excluded several relevant outcome papers particularly based on the defined age range. Many studies on adult patients include participants up until the age of 35. However, it is also a strength of the study that it focuses on treatment of adolescents and young adults, as in most studies young adults are combined with older adults, who have a longer treatment duration with presumably larger deteriorating effects, e.g., on functioning, and who might respond to treatment differently than patients at an early stage of the illness. It is a challenge also for research with youth that the developmental changes from early to later adolescence and adulthood occur rapidly meaning that it might cofound with the changes interpreted as a result from the intervention. In addition, even if limiting the age range to younger adults, it should be noted that conducting a psychological intervention to early adolescents might, and presumably should, differ from conducting the same intervention to adults. So, there might be within-group differences in the treatments relating to the age of participants that were not addressed in this study. In addition, the studies including patients at high risk for psychosis were excluded in case of a percentage over 50% for high-risk patients, as the review focuses on patients who fill the diagnostic criteria of psychotic disorders.

It should be acknowledged that since many of the studies implemented additional care together with the studied interventions, it is difficult to judge whether possible improvements can be contributed only to the interventions. Additionally, the main limitation of the included non-randomized studies was related to the confounding bias. In addition, we found only one non-controlled study meeting the inclusion criteria of our search, even if the search was not limited to controlled trials. This raises the question that there might be other clinical trials that were not identified in our search, even if the search string should have included them. In addition, the focus of this systematic review was to report the between group effects, and results from the effectiveness of each target intervention was not reported on different outcome domains, so deeper conclusions of the within-treatment effects cannot be drawn from this systematic review.

## Conclusion

Young people with psychotic disorders are likely to benefit from individual, family, or group psychological interventions such as cognitive, behavioral, or cognitive behavioral therapy, CRT, and other psychological interventions targeting psychosis, especially when including the elements of psychoeducation and involving closed ones to it. However, with regard to symptom reduction no treatment modality seems to clearly outperform other active treatments. It is important to notice that the main aspect in psychotherapy is not only to reduce symptoms, similarly to what medication does, but to provide an add on (72) treating various psychological functions (from neurocognitive functions to self-esteem) and relational functioning (73). Psychological interventions are often needed in order to help persons to maintain or improve their level of psychological and social functioning after the onset of psychosis (74). Even if continued documentation on efficacy and effectiveness on symptoms and functioning is important, at the same time it would be important to do more research on outcomes that reflect a change in understanding the causes or adaptation to the disorder, family relations, mentalization etc. to broaden the knowledge and move the focus to the next level (75). Focusing more on predictors and moderators of treatment outcome would help us to understand better what works to whom and why, enabling the improvement of more individualized treatment recommendations.

## Data Availability Statement

The original contributions presented in the study are included in the article/Supplementary Material, further inquiries can be directed to the corresponding author.

## Author Contributions

RU and SP conceived the original idea. VG developed the search strategy, with the assistance of SP and EV. VG and EV formed the eligibility criteria and planned the data extraction. VG, BM, and HL-S conducted the screening and data extraction process and drafted the first version of the manuscript. BM did the quality assessment. RU coordinated the overall COST initiative. SP, RU, and EV provided critical insights into the first version of the manuscript. All authors have approved and contributed to the final written manuscript with VG finalizing it.

## Conflict of Interest

The authors declare that the research was conducted in the absence of any commercial or financial relationships that could be construed as a potential conflict of interest.

## Publisher’s Note

All claims expressed in this article are solely those of the authors and do not necessarily represent those of their affiliated organizations, or those of the publisher, the editors and the reviewers. Any product that may be evaluated in this article, or claim that may be made by its manufacturer, is not guaranteed or endorsed by the publisher.
