# Supplementary material for: Psychological Interventions for Young People With Psychotic Disorders: A Systematic Review
Source: Front Psychiatry. 2022 Mar 24;13:859042. doi: 10.3389/fpsyt.2022.859042 (PMC8987205; doi:10.3389/fpsyt.2022.859042)
Supplement: Supplementary file 1 [file Data_Sheet_1.pdf]

Supplementary Material

1 Supplement File 1A. Search string used to identify relevant studies in PubMed.

((((((("psychotic disorders"[MeSH Terms] OR ("psychotic"[All Fields] AND "disorders"[All Fields]) OR "psychotic disorders"[All Fields] OR ("psychotic"[All Fields] AND "disorder"[All Fields]) OR "psychotic disorder"[All Fields]) OR "psychosis"[All Fields]) OR "psychoses"[All Fields]) OR ("schizophrenia"[MeSH Terms] OR "schizophrenia"[All Fields])) OR (("schizoaffective"[All Fields] AND "disorders"[All Fields]) OR "schizoaffective disorders"[All Fields] OR ("schizoaffective"[All Fields] AND "disorder"[All Fields]) OR "schizoaffective disorder"[All Fields])) OR schizophreniform[All Fields]) AND ((("psychotherapy"[MeSH Terms] OR "psychotherapy"[All Fields] OR "Psychotherapeutic treatment"[All Fields] OR "Psychotherapeutic treatments"[All Fields] OR "psycho-therapeutic treatment"[All Fields] OR "Psychotherapeutic intervention"[All Fields] OR "Psychotherapeutic interventions"[All Fields] OR "psychological therapy"[All Fields] OR "psychological therapies"[All Fields] OR "psychological treatment"[All Fields] OR "psychological treatments"[All Fields] OR "psychological intervention"[All Fields] OR "psychological interventions"[All Fields] OR "psychosocial therapy"[All Fields] OR "psychosocial therapies"[All Fields] OR "psychosocial treatment"[All Fields] OR "psychosocial treatments"[All Fields] OR "psychosocial intervention"[All Fields] OR "psychosocial interventions"[All Fields] OR "supportive therapy"[All Fields] AND "supportive therapies"[All Fields] OR "supportive treatment"[All Fields] OR "supportive treatments"[All Fields] OR "counseling"[MeSH Terms] OR ("counselling"[All Fields] OR "counseling"[All Fields]) OR "motivational interviewing"[All Fields] OR "psychoeducation"[All Fields] OR "psychoeducational"[All Fields] OR "psycho-education"[All Fields] OR "psycho-educational"[All Fields]) OR ("cognitive therapy"[All Fields] OR "cognitive therapies"[All Fields] OR "behavioural therapy"[All Fields] OR "behavioural therapies"[All Fields] OR "behavioral therapy"[All Fields] OR "behavioral therapies"[All Fields] OR "cbt"[All Fields] OR "psychoanalysis"[MeSH Terms] OR "psychoanalysis"[All Fields] OR "psychodynamic therapy"[All Fields] OR "psychodynamic therapies"[All Fields] OR "psychoanalytic therapy"[All Fields] OR "psychoanalytic therapies"[All Fields] OR "dynamic therapy"[All Fields] OR "dynamic therapies"[All Fields] OR "transference focused"[All Fields] OR "mentalization based"[All Fields] OR "metacognitive therapy"[All Fields] OR "metacognitive therapies"[All Fields] OR "interpersonal therapy"[All Fields] OR "interpersonal therapies"[All Fields] OR "interpersonal and social rhythm therapy"[All Fields] OR "schema therapy"[All Fields] OR "Schema-focused Therapy"[All Fields] OR "Schema-focused Therapy"[All Fields] OR "acceptance and commitment therapy"[All Fields] OR "acceptance based"[All Fields] OR "problem solving therapy"[All Fields] OR "problem solving therapies"[All Fields] OR "problem solving treatment"[All Fields] OR "problem solving treatments"[All Fields] OR "insight oriented therapy"[All Fields] OR "insight oriented therapies"[All Fields] OR "rational emotive"[All Fields] OR "solution focused therapy"[All Fields] OR "solution focused therapies"[All Fields] OR "family therapy"[All Fields] OR "family therapies"[All Fields] OR "family systems therapy"[All Fields] OR "parenting intervention"[All Fields] OR "parenting interventions"[All Fields] OR "parent management training"[All Fields] OR "group therapy"[All Fields] OR "group therapies"[All Fields] OR "mind-body therapies"[MeSH Terms] OR "mind body therapy"[All Fields] OR "mind body therapies"[All Fields] OR "art therapy"[All Fields] OR "art therapies"[All Fields] OR "dance therapy"[All Fields] OR "dance therapies"[All Fields] OR "music therapy"[All Fields] OR "music therapies"[All Fields] OR "play therapy"[All Fields] OR "play

therapies"[All Fields] OR "expressive therapy"[All Fields] OR "expressive therapies"[All Fields])) OR ("cognitive remediation"[All Fields] OR "cognitive training"[All Fields] OR "behavioral activation"[All Fields] OR "behavior activation"[All Fields] OR "behavioural activation"[All Fields] OR "applied behavior analysis"[All Fields] OR "applied behaviour analysis"[All Fields] OR "behavioral weight control"[All Fields] OR "behavioural weight control"[All Fields] OR "attention bias modification"[All Fields] OR (("attention"[MeSH Terms] OR "attention"[All Fields]) AND bias-modification[All Fields]) OR "exposure and response prevention"[All Fields] OR (exposure[All Fields] AND "response prevention"[All Fields]) OR "exposure therapy"[All Fields] OR "systematic desensitization"[All Fields] OR "eye movement desensitization reprocessing"[All Fields] OR "emdr"[All Fields] OR "psychology biofeedback"[All Fields] OR "hypnosis"[All Fields] OR "mindfulness"[All Fields] OR "relaxation"[MeSH Terms]))) AND (("adolescent"[MeSH Terms] OR "adolescent"[All Fields] OR "adolescents"[All Fields]) OR ("young adult"[MeSH Terms] OR "young adult"[All Fields] OR "young adults"[All Fields])) AND (Clinical Study[ptyp] OR Comparative Study[ptyp])

2 Supplement File 1B. Search string used to identify relevant studies in PsycINFO.

1. adolescen*.mp.

2. young adult*.mp.

3. 1 or 2

4. psychotherap*.mp. or exp PSYCHOTHERAPY/

5. Psychotherapeutic treatment*.mp.

6. Psychotherapeutic intervention*.mp.

7. Psychological therap*.mp.

8. Psychological treatment*.mp.

9. Psychological intervention*.mp.

10. Psychosocial therap*.mp. [mp=title, abstract, heading word, table of contents, key concepts, original title, tests & measures]

11. Psychosocial treatment*.mp.

12. Psychosocial intervention*.mp.

13. Supportive therap*.mp. [mp=title, abstract, heading word, table of contents, key concepts, original title, tests & measures]

14. Supportive treatment*.mp.

15. Counselling.mp.

16. exp COUNSELING/ or counseling.mp.

17. exp Motivational Interviewing/ or Motivational interviewing.mp.

18. exp PSYCHOEDUCATION/ or Psychoeducation*.mp.

19. 4 or 5 or 6 or 7 or 8 or 9 or 10 or 11 or 12 or 13 or 14 or 15 or 16 or 17 or 18

20. cognitive therap*.mp. or exp Cognitive Therapy/

21. Cognitive analytic therap*.mp. [mp=title, abstract, heading word, table of contents, key concepts, original title, tests & measures]

22. Behavioral therap*.mp. or exp Cognitive Behavior Therapy/

23. Behavioural therap*.mp. [mp=title, abstract, heading word, table of contents, key concepts, original title, tests & measures]

24. CBT.mp.

25. Psychoanalysis.mp. or exp PSYCHOANALYSIS/

26. Psychodynamic therap*.mp.

27. Psychoanalytic therap*.mp.

28. Dynamic therap*.mp. [mp=title, abstract, heading word, table of contents, key concepts, original title, tests & measures]

29. Transference focused.mp.

30. Mentalization based.mp.

31. Metacognitive therap*.mp. [mp=title, abstract, heading word, table of contents, key concepts, original title, tests & measures]

32. Interpersonal therap*.mp. or exp Interpersonal Psychotherapy/

33. (Interpersonal and social rhythm therap*).mp. [mp=title, abstract, heading word, table of contents, key concepts, original title, tests & measures]

34. exp Schema Therapy/ or Schema therap*.mp.

35. Schema-focused therap*.mp. [mp=title, abstract, heading word, table of contents, key concepts, original title, tests & measures]

36. "Acceptance and Commitment Therap*".mp. or exp "Acceptance and Commitment Therapy"/

37. Acceptance based.mp.

38. Problem solving therap*.mp. [mp=title, abstract, heading word, table of contents, key concepts, original title, tests & measures]

39. Problem solving treatment*.mp. [mp=title, abstract, heading word, table of contents, key concepts, original title, tests & measures]

40. exp Insight Therapy/ or Insight oriented therap*.mp.

41. exp Rational Emotive Behavior Therapy/ or Rational emotive.mp.

42. exp Solution Focused Therapy/ or Solution focused.mp.

43. Family therap*.mp. or exp Family Therapy/

44. Family systems therap*.mp. [mp=title, abstract, heading word, table of contents, key concepts, original title, tests & measures]

45. exp Family Intervention/ or exp Parent Training/ or Parenting intervention*.mp.

46. Parent management training.mp.

47. Group therap*.mp. or exp Group Psychotherapy/

48. exp Mind Body Therapy/ or Mind-Body Therap*.mp.

49. exp Art Therapy/ or Art Therap*.mp.

50. Dance Therap*.mp. or exp Dance Therapy/

51. Music Therap*.mp. or exp Music Therapy/

52. Play Therap*.mp. or exp Creative Arts Therapy/

53. exp Expressive Psychotherapy/ or Expressive therap*.mp.

54. 20 or 21 or 22 or 23 or 24 or 25 or 26 or 27 or 28 or 29 or 30 or 31 or 32 or 33 or 34 or 35 or 36 or 37 or 38 or 39 or 40 or 41 or 42 or 43 or 44 or 45 or 46 or 47 or 48 or 49 or 50 or 51 or 52 or 53

55. Cognitive remediation.mp.

56. Cognitive training.mp.

57. Behavioral activation*.mp.

58. Behavioural activation*.mp.

59. Behavior activation*.mp.

60. Behavioral weight control*.mp.

61. Behavioural weight control*.mp.

62. exp Behavior Modification/ or exp Behavior Analysis/ or Applied behavior analysis.mp.

63. exp Behavior Therapy/ or Applied behaviour analysis.mp.

64. Attention bias modification.mp.

65. "Exposure and response prevention*".mp.

66. exposure therap*.mp. or exp Exposure Therapy/

67. exp Systematic Desensitization Therapy/ or Systematic Desensitization.mp.

68. exp Eye Movement Desensitization Therapy/ or Eye movement desensitization reprocessing.mp.

69. EMDR.mp.

70. Psychology biofeedback.mp.

71. Hypnosis.mp. or exp HYPNOSIS/

72. Mindfulness.mp. or exp MINDFULNESS/

73. Relaxation.mp. or exp RELAXATION THERAPY/ or exp RELAXATION/

74. 55 or 56 or 57 or 58 or 59 or 60 or 61 or 62 or 63 or 64 or 65 or 66 or 67 or 68 or 69 or 70 or 71 or 72 or 73

75. 19 or 54 or 74

76. "Schizophrenia Spectrum and Other Psychotic Disorder*".mp.

77. psychotic disorder*.mp.

78. exp ACUTE PSYCHOSIS/ or exp CHRONIC PSYCHOSIS/ or exp PSYCHOSIS/ or psychosis.mp.

79. exp Schizophrenia/ or schizophreni*.mp.

80. exp SCHIZOAFFECTIVE DISORDER/ or schizoaffective.mp.

81. exp SCHIZOPHRENIFORM DISORDER/ or schizophreniform.mp.

82. exp Reactive Psychosis/ or reactive psychosis.mp.

83. psychoses.mp.

84. reactive psychoses.mp.

85. 76 or 77 or 78 or 79 or 80 or 81 or 82 or 83 or 84

86. 3 and 75 and 85

87. limit 86 to (("0200 clinical case study" or "0300 clinical trial" or "0400 empirical study" or 2100 treatment outcome) and (200 adolescence or 320 young adulthood))

3 Supplement File 2. Descriptions of the psychological interventions included in the systematic review under the defined treatment categories.

Cognitive/cognitive-behavioural/behavioural therapy

Social cognition and interaction training (SCIT)

Bartholomeusz et al. (28) investigated the outcome of treatment intervention by implementing social cognition and interaction training program (SCIT). In addition to remediating social cognition, SCIT has been proven to be successful in treating social functioning. The intervention is a group program, which consisted of 20 manual-based sessions (two hours weekly, for 10 weeks) and focused on three distinct aspects: (i) emotion recognition training while considering social context; (ii) recognizing attributional styles and understanding different scenarios; and (iii) integration of previously addressed skills into real-life situations. The program was used with young patients (16-26 years old) with first-episode psychosis and was consequently adapted for this age range.

Cognitive behavioral therapy for adolescents with psychosis (CBTpA)

Browning et al. (47) implemented two treatment interventions. The first treatment involved cognitive-behavioral therapy for adolescents with psychosis (CBTpA). This is a manual-based intervention comprising 10 sessions, which were delivered up to twice per week. Specific areas of psychotherapeutic work focused on the psychotic symptoms (coping, reappraisal, validity testing), affect, self-esteem, and on dealing with maladaptive and stigmatizing aspects of psychosis and mental health problems. The study implemented a version of the intervention that was adapted according to the age and the developmental stage of the participants (i.e., sessions were shortened to 30 mins and the location was flexible). The principle of the intervention was to normalize rather than pathologize aspects of the young person's presentation.

Family intervention for adolescents with psychosis (FIpA)

The second treatment intervention conducted in the Browning et al. study (47) was a family intervention for adolescents with psychosis (FIpA). This intervention involved five-hour long sessions with two co-therapists and was delivered over 4–10 weeks. The intervention had a psycho-educational component that focused on sharing information about psychosis, its causes, and possible treatment options with the family, with the goal to improve the family's understanding of the illness. In the terms of concrete areas of psychotherapeutic work, an emphasis was placed on helping the family to identify precipitating stresses and to plan strategies for coping with future difficulties.

Relapse prevention treatment (RPT) for first episode psychosis (FEP)

Gleeson et al. (33-34) reported on the combined individual and family-based CBT program for relapse prevention (RPT) in participants with first episode psychosis. The individual therapy intervention was provided biweekly during a seven-months period. The intervention consisted of five phases of therapy including assessment focusing on the extent of recovery and the risk of relapse (e.g., substance use, medication non-compliance, stressful life events, comorbid anxiety and depression), agreement on agenda of the therapy, acknowledgment of the risk of setbacks and possible solutions, identification of the early warning signs of relapse and formulation of a plan for

relapse prevention; the final (fifth) phase was optional and addressed issues of non-adherence to treatment and possible comorbidities (i.e., substance use, anxiety, depression).

The family part of the intervention was also manualized, based on CBT for schizophrenia and family interventions for first episode psychosis (FEP). The phases of family therapy involved assessment of family communication, burden and coping, psychoeducation regarding relapse risk, review of early warning signs of relapse, and documentation of a relapse prevention plan. When needed, intensive communication skills training and problem solving were also provided.

Helping young people early (HYPE) program + specialist first-episode psychosis treatment (SFET)

In Gleeson et al., (46) participants were treated according to the Helping Young People Early (HYPE) program, which was provided together with the specialist first-episode psychosis treatment (SFET). This program was developed as an early intervention program for young people with borderline personality disorder (BPD). It was a team-based intervention model that integrated time-limited cognitive analytic therapy (CAT) and elements of psychoanalytic object relations theory used with persons with complex and relational disorders, particularly BPD; additionally, case management and general psychiatric care were also provided. The main focus of the intervention was the individual's problematic relationship patterns and the thoughts, feelings, and behavioral responses, which may have resulted from such patterns. Participants had approximately 16 (up to one hour long) sessions conducted by two therapists.

Cognitively oriented psychotherapy for early psychosis (COPE)

The intervention conducted by Jackson et al. (35-37) involved four (manualized) phases: engagement, assessment, adaptation, and secondary morbidity. The agenda in COPE included psychoeducation, stigma, and identity issues with a focus on the patient's problems with motivation and confidence. The intervention was intended to provide a minimum of 15 (40 minutes) sessions, delivered on a flexible basis (once per week to biweekly) in out-patient service.

Active cognitive therapy for early psychosis (ACE)

Jackson et al. (38) and Allott et al. (39) implemented Active cognitive therapy for early psychosis (ACE) that was provided for a maximum of 20 sessions (approximately 45 mins) over 14 weeks. Therapy sessions were delivered flexibly across a range of settings (i.e., participant's own home, neutral location, treatment center) with the therapist deciding on the timing and duration of sessions. The main focus was on the presentation of psychotic and non-psychotic complaints followed by a formulation of the relationship between such complaints and the participant's life history. If positive psychotic symptoms were present and distressing, they were considered the second-highest priority; this was followed by co-morbidities, negative symptoms, issues of identity, and relapse prevention. All participants also received case management, medical assessment, and other services from the treatment center.

In-patient psychosocial and behavioral family intervention (IPFI)

In the study conducted by Lenior et al. (40), Linszen et al. (41-43), and Nugter et al. (44), participants in the treatment group received behavioral family intervention together with the standard psychosocial intervention (SI). This family treatment was based on behavioral family management approach but adapted for the clinic- (rather than home-) based context. The main content of the

therapy sessions involved psychoeducation, communication training, and problem-solving techniques. Treatment lasted for 12 months comprising 18 sessions (held biweekly during the first five months, and once a month for the remaining seven months). A similar approach was also used in Gleeson et al.[33-34] in the family part of the intervention with the focus on relapse prevention.

Managing Adolescent first episode Psychosis (MAPS)

In Morrison et al. (45), participants received a combination of individual CBT and (if consented) family intervention. Individual treatment was based on an integrative cognitive model, which involved themes such as identifying problems and agreeing on goals, factors leading to the development of first-episode psychosis, and relapse prevention. The family intervention was based on a behavioral model and involved aspects such as psychoeducational work, provision of normalizing information with recovery-oriented information, problem-solving, and relapse prevention planning. Treatment comprised 26 individual CBT and up to six sessions of family intervention, delivered once per week (for CBT) and monthly (for family intervention) over a 6-month period; additionally, participants received four booster sessions after the treatment period.

Group cognitive-behavioral treatment of auditory hallucinations

Treatment in Newton et al. (48) was based on cognitive-behavioral therapy delivered in a group setting with a protocol that included the main aims of the sessions, examples of interventions, model responses from the therapists, and was concluded with a final follow-up session. Sessions involved themes such as sharing of information about the voices, models of psychosis, models of hallucinations, effective coping strategies, improving self-esteem, and an overall model of coping with voices.

The Graduated Recovery Intervention Program (GRIP)

Penn et al. (32) investigated an individual cognitive-behavioral therapy program made to improve the functioning of persons with an initial episode of psychosis. The treatment involved a maximum of 36 sessions, which were provided on a weekly basis. The first 12 sessions were standardized and focused on: psychoeducation, processing the illness, goal setting, illness management, relapse prevention, and psychoeducation about substances. Additionally, a patient was asked to identify an ‘indigenous supporter’ (a friend, relative, or support person) who would assist the patient with homework or helped with a goal that had been set. The therapist (clinician) periodically also communicated with the identified supporter. After completing the first 12 sessions, the patient and therapist jointly assessed the progress and decided whether additional treatment was necessary.

Cognitive remediation therapy

Cognitive remediation therapy / training programs

The Cognitive Remediation Therapy (CRT) provided in Puig et al. (58) on an individual basis twice a week (in total 40 sessions) targets flexibility in thinking, information-set maintenance, executive functions for memory controll and planning, and schema formation. Comparable to the CRT approach offered in Wykes et al. (56) – also for 40 sessions, but three times per week - a therapy manual guides the modules for practicing remembering processes, complex planning or information

processing and problem solving. Both, the CRT in Wykes et al. (56) lasting for three months and in Puig et al. (58) provided for five months, were accompanyied by antipsychotic medication.

The Cognitive Remediation training program presented in Ueland and Rund (51-52) includes 30 hours of individual training consisting of four modules: cognitive differentiation, attention, memory and social perception. In addition, all patients received psychoeducational treatment, parent seminars, problem-solving sessions, milieu therapy and network groups during the six months of treatment, and antipsychotic medication was prescribed in about 80% of the patients.

Cognitive Remediation Programme (NEUROCOM)

The remediation programme NEUROCOM in Østergaard Christensen et al. (59) combined cognitive training, offered one hour (twice per week) for 16 weeks, with an early intervention service (OPUS-EIS). While the cognitive training part of NEUROCOM targeting domains of attention, executive functions and learning/memory is comparable to average computerized cognitive trainings (as, e.g., COGNIsoft), a competence dialogue one hour every two weeks is added in NEUROCOM. The competence dialogues addressed work and social competencies, as well as self-experienced cognitive competencies (e.g., attention). The total remediation programme consisted of 2.5 hours per week and was conducted additionally to the OPUS-EIS. The latter includes patient and family psychoeducation, medication, social skills training and individual vocational support, aiming mainly at coping with the illness.

The Cognitive Remediation program for patients with Schizophrenia (RECOS), The Autobiographical Reminiscence Therapy (REMAu) and The Mindfulness-based Cognitive Therapy (MBCT)

In Lalova et al. (57), three different types of cognitive remediation programs were compared. The Cognitive Remediation program for patients with Schizophrenia (RECOS) targets subjects' cognitive skills relevant to everyday life (reasoning, verbal memory, visuo-spatial, working memory, and selective attention) in five modules within individual sessions once a week with additional homework (psycho-educative part). The Autobiographical Reminiscence Therapy (REMAu), also provided on an individual basis, targets more the persons' semantic knowledge and episodic (incl. autobiographical) memories. The Mindfulness-based Cognitive Therapy (MBCT), conducted in a group with 8-10 patients, targets patients' perceptual experiences, attributions and attitudes, and includes meditation and breathing exercises. All three remediation programs consisted of 12 sessions (one hour) and 30min home-exercises, and were combined with atypical antipsychotic medication.

Computer-assisted cognitive training programs

In Corbera et al. (49), the standardized CR interventions based on the CR manual-guided moduls designed by Wykes et al. (56) targetting basic neurocognitive skills (attention, working memory, verbal and non-verbal memory, executive functions and language processing) on different levels of difficulty, were computerized and reached up to a potential maximum of 100 hours of training. Computerization made a drill-and-practice design possible. Patients additionally received antipsychotic medication, vocational counseling, psychoeducation, goal-setting groups and case-management on a three times per week basis.

A conceptually similar computer-assisted CR program (CACR), "Captain's Log", was offered in Urben et al. (53), Pihet et al. (54) and Holzer et al. (55) targeting attention, memory, concentration,

processing speedvisual-spatial, visual-motor functioning, response inhibition, categorization, and conceptualization, again with increasing difficulty. In the study 16 individual 45min sessions twice per week were offered, and the majority, but not all patients, also received antipsychotic medication. Trainers selected the tasks, provided the direction and level of dificulty, and gave support by giving encouragement and positive feedback.

Dang et al. (50) offered iPad assisted cognitive training (CT) one hour per day/ five times per week assisted by nurses, which mainly targeted working memory. No further social-psychiatric treatment, but antipsychotic medication was provided. Similar to the iPad assisted cognitive training, Fisher et al. (29) and Puig et al. (30) provided a Neuroplasticity-based Auditory Training (AT) via laptop computer targeting auditory and verbal working memory via fostering speed and accuracy of auditory information processing. Patients participated at home one hour daily / five days per week and were coached via telephone 1-2 times per week. They received monetary rewards per training hour and had a mean duration of 34.65 h training time. Additionally, psychoeducation, antipsychotic medication and psychotherapy were offered.

Other psychological interventions

Early detection plus integrated care (EDIC)

In Lambert et al. (31), the target intervention was early detection plus integrated care (EDIC) aiming to reduce duration of psychosis and improve the quality of care. The methods integrated into the treatment were (i) mobile early detection, (ii) acute and long-term treatment, (iii) case management (planning, organization, and monitoring of specialized interventions within the network (e.g., social therapy, diagnosis-specific psychoeducation, trauma and substance use therapy, metacognitive training, cognitive remediation, social skills training, vocational therapy), (iv) pharmacotherapy, and (v) intensive individual psychotherapy, including CBT, psychodynamic, and/or family therapy. The average number of sessions during the 1-year treatment period was 184.4 (SD 103.5), which corresponds in average to 3.5 therapeutic sessions per week.

Extended early intervention (EEI)

In Chang et al. (63), the studied intervention was extended early intervention (EEI). Specialised early intervention was continued in the form of an additional year of case management focusing specifically on functional enhancement by assisting participants to re-establish supportive social networks, resume leisure pursuits and return to work. Also continuous supportive care, psychoeducation, and coping and stress management were delivered to family caregivers. The participants received on average 16 intervention sessions, each lasting for a minimum of 30 min with either clinical assessment conducted or psychosocial treatment delivered by the case manager, over the 1 year treatment period.

Integrative psychoeducational family treatment (IPEF)

Rund (60), and Rund et al. (61-62) reported on integrative psychoeducational family treatment (IPEF). The patients were initially hospitalized for a periode ranging from some months to one year, and an intensive rehabilitation process lasting up to two years from the beginning of the hospitalization was started when the patient was discharged from the ward. Central elements of the treatment programme were parent seminars, problem-solving sessions, milieu therapy and networks.

During the hospitalization, family treatment sessions were conducted every second week, and the parents took part in a whole day parent seminar. Furthermore, problem-solving sessions were started during this phase for some patients, which were continued during the outpatient period where the problem solving sessions took place once a month in the patient's home. Parents or relatives also took part in additional seminars. The frequency of the family treatment sessions were reduced to once every second month towards the end of the treatment. The parent seminars focused on the resources of the family, offered psychoeducation on psychoses and general advice to parents, and aimed to reduce the guilt and shame they might have. The problem-solving sessions offered to the family were structured and consisted of several steps towards a solution of defined problems. Also the patients' networks, such as persons from the school environment were involved in the treatment to compose an individual rehabilitation program for each patient.

Psychoeducational group intervention (PGI)

In Calvo et al. (66-67), the target intervention was psychoeducational group intervention (PGI) that included parallel, structured and specific psychoeducational group interventions for adolescents and their families as an add-on intervention to treatment as usual. The treatment consisted of 12 sessions of 90 minutes each, once every 15 days. Sessions were structured, and the patients and their families also received written material. Groups specifically focused on problem-solving strategies to manage daily life difficulties associated with the disease to mitigate crises and prevent relapses. Content and group structure were the same in the adolescents and parents' version of the program.

Structural group therapy (SGT)

She et al. (65) reported on the outcomes of 12-session structural group therapy (SGT) conducted in an inpatient adolescent hospital unit. Each group had ten participants. The program was a preliminary design based on designs and resources of adolescent group therapy strategies and methodologies described in other studies, that was reviewed and revised based on recommendations from an expert panel. The sessions were offered twice a week for six weeks. Each session lasted 60 minutes, and treated different themes (e.g., emotions, roles, self-cognition, strenghts) and used types of activities (e.g., games, role play, handwork, biography).

Group psychosocial treatment (PST)

Koren and Stepunina (64) studied the effectiveness of group psychosocial treatment (PST) based on the conceptual principles of psychosocial rehabilitation. The program was conducted in groups of six to eight patients, and each group had a 120 minute session once a week over 12-week period. The treatment included training of communication skills based on a psycho-educational approach with elements of motivational training and training to cope with residual symptoms of illness, along with the use of art therapy methods. In the sessions, a flexible, individualized approach to each participant was used to allow utilizing the mechanisms of group interactions to solve ongoing sociotherapeutic tasks taking into account individual characteristics, family situation, and the level of social adaptation.

Supplement File 3. Risk of bias and quality assessment for included studies.

	Screening for all (+/±/-)		Screening for quantitative randomized controlled trials (RCTs) (+/±/-)					Screening for quantitative non-randomized trials (+/±/-)				
Study reference	Were there clear research questions?	Did the collected data allow to address the research questions?	Was randomization appropriately performed?	Were the groups comparable at baseline?	Was there complete outcome data?	Were outcome assessors blinded to the intervention provided?	Did the participants adhere to the assigned intervention?	Were the participants representative of the target population?	Were measurements appropriate regarding both the outcome and intervention?	Was there complete outcome data?	Were the confounders accounted for in the design and analysis?	During the study period, was the intervention administered as intended?
**CT/CBT/BT**												
Bartholomeusz et al., 2013 (28)	+	+						+	+	-	-	+
Browning et al., 2012 (47)	+	+						+	+	+	-	+
Gleeson et al., 2009 (33) & 2013 (34) (RCT)	+	+	+	+	+	+	+					
Gleeson et al., 2012 (46) (RCT)	+	+	+	+	-	-	-					
Jackson et al., 1998 (35) & 2001 (36) & 2005 (37)	+	+						+	+	-	-	+
Jackson et al., 2008 (38); Allott et al., 2011 (39) (RCT)	+	+	+	+	+	+	+					
Lenior et al. 2002 (40); Linszen et al. 1996 (41) & 1997 (42) & 2001 (43); Nugter et al. 1997 (44) (RCT)	+	+	+	+	+	+	+					
Morrison et al., 2020 (45) (RCT)	+	+	+	+	±	±	+					
Newton et al., 2005 (48)	+	+						+	+	+	-	+
Penn et al., 2011 (32) (RCT)	+	+	+	+	±	+	-					

**CRT**												
Corbera et al., 2017 (49) (RCT)	+	+	±	+	+	+	+					
Dang et al., 2014 (50) (RCT)	+	+	±	+	+	±	+					
Fisher et al., 2015 (29); Puig et al., 2020 (30) (RCT)	+	+	+	+	-	+	-					
Lalova et al., 2013 (57) (RCT)	+	+	±	+	+	±	+					
Østergaard Christensen et al., 2014 (59) (RCT)	+	+	+	+	+	+	+					
Puig et al., 2014 (58) (RCT)	+	+	+	+	-	+	-					
Ueland & Rund, 2004 (51) & 2005 (52) (RCT)	+	+	+	+	+	±	+					
Urben et al., 2012 (53); Pihet et al., 2013 (54); Holzer et al., 2014 (55) (RCT)	+	+	+	+	-	+	-					
Wykes et al., 2007 (56) (RCT)	+	+	+	+	±	±	+					
**OTHER TREATMENTS**												
Calvo et al., 2014 (66) & 2015 (67) (RCT)	+	+	+	+	±	+	-					
Chang et al., 2015 (63) (RCT)	+	+	+	+	+	+	+					
Koren & Stepunina, 2015 (64) (RCT)	+	+	±	+	+	±	+					
Lambert et al., 2017 (31)	+	+						+	+	-	-	+
Rund 1994a (60); Rund et al. 1994b (61) & 1995 (62)	+	+						+	+	+	-	+
She et al., 2016 (65) (RCT)	+	+	+	+	±	+	+					

† Yes (+), unclear (±), no (-).
